# Seeding-competent early tau multimers are associated with cell type-specific transcriptional signatures

**DOI:** 10.1007/s00401-025-02869-4

**Published:** 2025-04-04

**Authors:** Rahel Feleke, Simona Jogaudaite, Elisavet Velentza-Almpani, Leung Yeung-Yeung, Daniel Clode, Jeong Hun Ko, Ben Shin, Steve Matthews, Maria Otero-Jimenez, Marcelina J. Wojewska, Sandra Gray-Rodriguez, Sarah J. Marzi, Maxwell P. Spires-Jones, Tara L. Spires-Jones, Michael R. Johnson, Javier Alegre-Abarrategui

**Affiliations:** 1 Department of Brain Sciences, Imperial College London, Hammersmith Hospital, London, W12 0NN UK; 2 UK Dementia Research Institute at King’s College London, London, SE5 9RT UK; 3 Department of Basic and Clinical Neuroscience, Institute of Psychiatry, Psychology and Neuroscience, King’s College London, London, SE5 9RT UK; 4 Centre for Discovery Brain Sciences and UK Dementia Research Institute, The University of Edinburgh, Edinburgh, EH8 9JZ UK

**Keywords:** Alzheimer's disease, Tau multimers, Early pathology, Proximity-ligation assay, Seeding, Single-nucleus RNA sequencing

## Abstract

The initial molecular alterations of Alzheimer’s disease (AD) are unknown. Established AD is characterized by profound structural and transcriptional alterations in the human brain, with the hallmark neuropathological features being beta-amyloid (Aβ) accumulation in senile plaques and hyperphosphorylated fibrillar tau in neurofibrillary tangles (NFTs). Previous evidence indicates that tau multimerization into small aggregates is one of the earliest molecular alterations, anticipating the accumulation of hyperphosphorylated tau in NFTs. In this study, we investigated the seeding capacity of these early small tau multimers and the transcriptional changes associated with them, aiming to unveil early pathogenic processes in AD-type tau pathology. Early tau multimers visualized with tau proximity ligation assay (tau-PLA) in the post-mortem temporal cortex demonstrated high seeding activity detected by real-time quaking-induced conversion (RT-QuIC) assay and induction of aggregates in a tau biosensor cell line. Using single-nucleus transcriptomics, we showed that brain tissue harboring seeding-competent early tau multimers, but without significant NFT pathology, is associated with substantial gene expression alterations across diverse cell types when compared to control tissue lacking either multimers or NFTs. Differentially expressed genes, such as *APP*, *MAPT*, and *PSEN1,* exhibited significant enrichment of AD heritability in up-regulated genes within excitatory neurons, astrocytes, and oligodendrocytes. Pseudotime analysis exposed a positive correlation between the progression of tau pathology and the expression of genes marking reactive astrocytes. In summary, our results support the hypothesis that seeding-competent tau multimerization may initiate AD-type tau pathology cascades before the accumulation of tau in NFTs. This research contributes valuable insights into the early molecular events associated with AD, with implications for future diagnostic and therapeutic strategies.

**Supplementary Information:**

The online version contains supplementary material available at 10.1007/s00401-025-02869-4.

## Introduction

Alzheimer's disease (AD) is a progressive neurodegenerative disorder characterized by the presence of two pathological protein aggregates within the brain—beta-amyloid (aβ) accumulating into senile plaques and phosphorylated tau protein accumulating into neurofibrillary tangles (NFTs) [22]. Traditionally seen as the main causes of AD, these lesions have been the focus of research, but the lack of effective treatments has prompted the exploration of other factors. Increasing attention is being given to tau oligomers, which are small, soluble aggregates of abnormally altered tau proteins. In vitro studies show that, unlike tau monomers, tau oligomers cause synapse loss, elevate calcium levels, and disrupt neurotransmitter-release balance [65]. In addition, injecting tau oligomers into mice leads to more severe mitochondrial abnormalities and synaptic dysfunction compared to NFTs [44]. In human brain studies, tau oligomers have been detected in regions without tangles and are correlated with cognitive decline, highlighting their detrimental effects on brain function [6, 44, 51, 56].

Emerging research indicates that tau self-interaction is one of the earliest events in AD progression, with early tau multimers playing a pivotal role in the recruitment and aggregation of native tau, a process known as seeding. Studies have demonstrated that not only insoluble large tau aggregates, but also soluble tau species can induce misfolding and aggregation of tau monomers [21, 30, 33, 49]. Furthermore, these small, soluble tau aggregates have been shown to propagate trans-synaptically from affected to unaffected brain regions, causing misfolding of native tau proteins within recipient neurons [20]. To gain deeper insights into the behavior of early tau multimers, researchers have employed seed amplification assays, such as real-time quaking-induced conversion (RT-QuIC), which offer heightened sensitivity and earlier detection of tau pathology compared to conventional techniques like immunohistochemistry (IHC) [52]. These assays have detected tau seeding activity well before the earliest AD stages, including in cognitively normal and young individuals, suggesting that tau multimerization may precede AD onset [40, 48].

However, a deeper understanding of the timing and progression of tau seeding relative to AD onset is hindered by the significant challenges in visualizing small tau aggregates in their native brain environment. In the last two decades, AT8-IHC has been the gold standard for the pathological diagnosis and staging of tau AD pathology, with the AT8 antibody capturing hyperphosphorylated fibrillar lesions [2, 10]. Further tau antibodies have been developed like the phospho-tau antibodies AT180 (pThr231), PHF-1 (pSer396/pSer404), T217 (pThr217), and S422 (pSer422), all of them being reported to capture phosphorylated events that occur early in the development of the AD-type tau pathology [5, 26, 45], and the conformational antibodies MC1 and Alz50 detecting early misfolded tau [2, 5, 10, 23, 26, 45, 55]. However, despite the proliferation of these antibodies, their validation remains an ongoing process.

To overcome the limitations inherent in IHC, particularly its reduced specificity and sensitivity in detecting small tau aggregates, our laboratory has developed a novel tau proximity ligation assay (tau-PLA) [9]. This innovative assay is uniquely capable of in situ histological visualization of tau aggregates composed of two or more molecules, while effectively excluding monomers. By performing tau-PLA, our research has revealed that tau multimerization appears extensively from early pre-symptomatic Braak stages in previously unrecognized medial temporal/hippocampal areas, well before any tau pathology can be detected by AT180-, AT8-, and MC1-IHC [9]. However, a key remaining question is whether the interactions between tau proteins in these early stages indicate the onset of pathological changes that ultimately result in AD.

In this study, we investigated the role of tau multimers in AD-type tau pathology by assessing the seeding capacity of small tau multimers and the transcriptional changes associated with their presence in the human brain. Expanding on our previous work on early tau pathology in the hippocampal regions [9], this study extends its analysis to the temporal and occipital cortices of 67 post-mortem cases, representing a comprehensive range of Braak stages I to VI, as well as Braak 0 brains. Utilizing tau-PLA, we detected tau multimerization that maps onto the Braak tau anatomical pathway, but precedes neurofibrillary lesions such as NFTs not only in the hippocampus but also in the temporal lobe convexity and occipital cortices. Our results highlight the temporal cortex, situated in an intermediate position in the Braak tau pathway, as a window to study alterations, including seeding capacity and gene expression changes. To unravel the early alterations associated with tau aggregation, we categorized cases with available frozen tissue based on the presence or absence of detectable tau pathology within the temporal cortex, namely: those without tau pathology measured by negative tau-PLA and AT8-IHC (Double-Negative); those with tau multimers identified through tau-PLA with minimal tangles as detected by AT8-IHC (Intermediate); and those exhibiting both tau multimers identified by tau-PLA and a substantial number of tangles detected by AT8-IHC (Double-Positive). Through this classification, we identified significant histological and transcriptomic alterations in the brain occurring before the formation of tau tangles and the clinical manifestation of AD-type tau pathology. Our findings suggest that seeding-competent tau multimerization is an early event in the brain's progression toward AD, with important implications for the design of transcriptomic and neuropathological studies of AD.

## Materials and methods

### Animal tissue

*MAPT* knock-out (KO) mice missing exon 1 in the *MAPT* gene after replacement with the neomycin-resistant cassette were provided by Marian Vargas-Caballero. Two-month-old *MAPT* KO mice were deeply anesthetized and subjected to transcardiac perfusion with phosphate-buffered saline (PBS). The brain was then extracted, and the right hemisphere was fixed in 4% paraformaldehyde (PFA) in PBS at 4 °C for 24 h. Subsequently, the fixed tissue was embedded in paraffin and sectioned using a microtome. The left hemisphere of the brain was stored at − 80 °C for further biochemical analysis.

### Human tissue

5 µm thick sections of formalin-fixed paraffin-embedded (FFPE) tissue encompassing temporal and occipital cortices of 67 post-mortem brains were prepared. As classified by NFT Braak stages (refer to Supplementary Table 1a), these samples span the entire spectrum of AD progression and were utilized for IHC and tau-PLA. To deepen our knowledge of the pathophysiological changes of incipient tau pathology, we focused on further analyses in the temporal cortex (middle temporal gyrus) corresponding to an intermediate position in the Braak tau pathway. We selected 16 brains from our cohort with fresh frozen tissue from the temporal cortex available. These samples were obtained from individuals with no clinically manifest neurological disease. The Braak stages of these 16 cases ranged from 0 to IV, as elaborated in Supplementary Table 1b*.* Fresh frozen samples were homogenized in ice-cold PBS (10% w/v) using a Tissue Ruptor II with disposable probes (Qiagen) and used for seeding assays and single-nucleus RNA sequencing (snRNA-seq). All samples were supplied by the Multiple Sclerosis and Parkinson's Tissue Bank of Imperial College London and The Oxford Brain Bank. Human post-mortem tissue was used according to The Oxford Brain Bank’s Research Ethics Committee Approval Ref. No. 15/SC/0639 (South Central—Oxford C Research Ethics Committee) and the Multiple Sclerosis and Parkinson’s Tissue Bank’s Research Ethics Committee Approval Ref. No. 08/MRE09/31 + 5 (Wales Research Ethics Committee).

### Immunohistochemistry

Human and animal FFPE tissue sections were heated at 70 °C for 45 min, followed by dewaxing in xylene and rehydration using graded alcohols (100–70%). To inhibit endogenous peroxidase activity, hydrogen peroxide treatment was applied. Antigen retrieval was performed by microwave heating with citrate buffer (pH 6.0) for a total of 10 min for certain antibodies (AT180, PS422, PT217), while no treatment was required for others (AT8, MC1, ALZ50, PHF-1). After blocking non-specific binding sites with 10% normal goat serum (S-1012-50, Vector Labs) or 10% normal horse serum (S-2000-20, Vector Labs) in TBS-0.1% Triton X-100 for 1 h at room temperature, the sections were incubated overnight at 4 °C with primary antibodies, including AT8 (MN1020, Innogenetics, 1:500), AT180 (MN1040, ThermoFisher Scientific, 1:1000), PS422 (44-764G, ThermoFisher Scientific, 1:500), PT217 (44-744, ThermoFisher Scientific, 1:1000), MC1, ALZ50, and PHF-1 (all kindly provided by Peter Davies, The Feinstein Institute for Medical Research, Manhasset, NY, at dilutions of 1:100, 1:100, and 1:1000, respectively). The following day, slides were incubated with biotinylated goat anti-mouse or anti-rabbit IgG secondary antibody (Jackson Immunoresearch) for 1 h at room temperature. After washing, Vectastain ABC reagents (Vector Labs) were applied to the sections. Slides were washed once again, and the detection was performed using a 3,3-diaminobenzidine (DAB) substrate according to the manufacturer's protocol. The slides were counterstained with Mayer's hematoxylin solution (MHS1, Sigma-Aldrich), dehydrated using graded alcohols (70–100%) and xylene, and finally mounted with a DPX mounting reagent.

### In situ tau-proximity ligation assay (tau-PLA)

The tau-PLA was conducted as previously described by us [9], using Duolink PLA kits (DUO92012 Sigma-Aldrich) following the manufacturer's instructions. Tau conjugates were prepared according to the Duolink PLA Probemaker protocol. The tau5 antibody (ab80579, Abcam) was used in conjunction with the Duolink PLA Plus or Minus kits (DUO92009 and DUO92010, respectively, Sigma-Aldrich) to generate the conjugates. Dewaxing of samples was performed as previously described, involving heating, dewaxing in xylene and histoclear, rehydration in graded alcohols (100–70%), blocking of endogenous peroxidase with 10% H_2_O_2_ for 1 h at room temperature, and antigen retrieval using microwave heating in citrate buffer (pH 6.0) for 10 min. Samples were incubated with Duolink blocking solution for 1 h at 37 °C and then with tau conjugates diluted in Duolink PLA diluent (1:250) overnight at 4 °C. After washing with TBS-0.05% Tween 20, the samples were incubated with Duolink ligation stock and ligase for 1 h at 37 °C. Following washes, incubation with Duolink amplification stock and polymerase for 2.5 h at 37 °C was performed. Tissue sections were then washed and incubated with a Duolink detection solution for 1 h at room temperature. After washing, the samples were incubated with the Duolink substrate reagent for 20 min at room temperature. Subsequently, the samples were counterstained with hematoxylin for 5 min, dehydrated using graded alcohols (70–100%) and xylenes, and finally mounted with a DPX mounting reagent.

### Imaging and neuropathological analysis

For imaging analysis of the stained sections, an Aperio-Scanscope with a 40× objective was utilized. Three representative images were blindly captured from the analyzed brain regions, and neuropathological analysis was performed using ImageJ software. During the processing phase, the images were thresholded, and the number of particles covering the total area was quantified. Particle size was adjusted to ranges of 12.5–100 µm^2^ for large perikaryal lesions, primarily representing NFTs, and 1.5–3.5 µm^2^ for the diffuse tau-PLA signal and IHC labeling small structures, as previously described [9]. The mean value for each sample and brain region was calculated and analyzed. Semi-quantitative analysis was conducted by blindly selecting three representative images for each sample and brain region based on a semi-quantitative scale ranging from 1 to 6 (refer to Supplementary Fig. 1a). GraphPad Software was used for statistical analysis and group comparison. One-way ANOVA with Dunnett's post hoc tests was performed, with statistical significance defined as *p* < 0.05.

### Purification of recombinant τ306 fragment

The τ306 tau fragment, encompassing residues 306–378 of the full-length human tau isoform htau40, with a point mutation at residue 322 (cysteine to serine) and a stop codon at the C-terminal residue 379, was obtained from the tau construct kindly provided by Byron Caughey. The cloning cassette was inserted into the pET-28a bacterial vector immediately after the 5' N-terminal poly-histidine tag and thrombin site using GenScript's CloneEZ seamless cloning technology [59]. Protein purification was performed following a previously described protocol [59]. Briefly, the τ306 fragment was expressed in the BL21(DE3) strain of *Escherichia coli* using the Overnight Express Autoinduction method [59]. The cells were harvested by centrifugation at 3273×*g* for 35 min at 4 °C, resuspended in Buffer A (5 mM imidazole, 10 mM Tris, pH 8.0, 500 mM NaCl), and subjected to sonication for 3 min (three cycles of 45 s sonication followed by a 15 s pause) at a power setting of 25%. The cell lysates were then centrifuged at 10,000×*g* for 1 h at 4 °C, and the supernatant was retained and filtered through a 0.45 μm syringe filter before proceeding to purification using a His-Trap FFTM nickel column (17-5255-01, GE Healthcare). To eliminate contaminants, the column was washed with 13 and 21% Buffer B (200 mM imidazole, 10 mM Tris, pH 8.0, 500 mM NaCl). The τ306 fragments were subsequently eluted with a linear gradient from 21% Buffer B to 100% Buffer B over 8 column volumes. Fractions of 2 mL were collected during the elution peak of the τ306 fragment, and each fraction was supplemented with 2 μL of 2 M dithiothreitol (DTT) to achieve a final concentration of 2 mM. Acetone precipitation of the τ306 fractions was then performed by adding 4 volumes of ice-cold acetone and incubating overnight at 4 °C. The precipitant was centrifuged at 10,000×*g* for 20 min at 4 °C and washed with 10 mL of acetone containing 2 mM DTT. After discarding the acetone, each pellet was dissolved in 2 mL of elution buffer (8 M guanidine hydrochloride in PBS). Desalting of the τ306 fragment was carried out using PD-10 desalting columns (GE Healthcare 17-0851-01). A spectrophotometer was used to confirm the removal of guanidinium chloride. The concentration of the τ306 fragments was adjusted to 0.75 mg/mL in 1X PBS, pH 7.0, and the samples were stored at -80 °C until further use.

### Recombinant τ306 fragment assembly

A solution of 1 mg/mL τ306 fragment was subjected to shaking at 37 °C and 250 rpm for 360 h in 10 mM HEPES (pH 7.0), 400 mM NaCl, and 40 µM Heparin (Sigma) to induce aggregation, following the protocol described by Kraus et al. [40]. Prior to use, the τ306 fragment was filtered through an Amicon Ultra-0.5 Centrifugal Filter Unit with a 100 kDa molecular weight cut-off (Sigma) to remove any aggregated fragments. The resulting τ306 aggregates were stored at − 80 °C until further use.

### Transmission electron microscopy

For transmission electron microscopy (TEM) analysis, 10 µL of 1 mg/mL τ306 aggregates were applied to carbon-coated TEM grids (TAAB) that had undergone glow discharge. The grids were incubated for 2 min at room temperature, followed by staining with 2% uranyl acetate for 10 s. The grids were then dried immediately and stored at room temperature, following the procedure previously described by Bengoa et al. [9]. TEM images were acquired using a FEI Tecnai 12 TEM microscope operating at 120 kV, equipped with a Gatan US1000 camera.

### Tau real-time quaking-induced conversion (RT-QuIC) assay

The tau RT-QuIC assay was performed according to the previously described methods by Saijo et al. and Kraus et al. [40, 59]. The τ306 tau substrate was thawed at room temperature and filtered using an Amicon 100 kDa molecular weight cut-off filter column (UFC510024, Millipore) at 3300×*g* for 5 min at room temperature to remove any aggregated τ306 species. The tau RT-QuIC reaction buffer consisted of 10 mM HEPES (pH 7.4), 400 mM NaCl, 40 μM heparin, 10 μM Thioflavin (ThT), and 0.1 mg/mL τ306. Brain homogenate samples (listed in Supplementary Table 1b) were diluted in a sample diluent buffer containing 10 mM HEPES (pH 7.4), 0.53% tau KO mouse brain homogenate, and 1 × N-2 supplement (Gibco), to a final concentration of 1 × 10^–1^. The inclusion of tau KO mouse brain homogenate and N-2 supplement in the diluent sample buffer was essential to prevent the spontaneous aggregation of the τ306 fragment. A 2 μM dilution of brain homogenate was transferred into triplicate wells of a 96-well plate, with a final volume of 100 μL per well. The plate was sealed and incubated at 42 °C in a BMG Omega FLUOStar plate reader, with intermittent shaking (1 min of orbital shaking at 500 rpm followed by 14 min of rest). ThT fluorescence readings (excitation: 450 ± 10 nm, emission: 480 ± 10 nm, bottom read) were recorded every 15 min. In addition to brain homogenates, postmortem cerebrospinal fluid (CSF) samples were also analyzed. Due to limited availability, only three CSF samples from the Double-Negative group and three from the Intermediate group were included (Supplementary Table 1b). The same protocol was followed, with 5 μL of undiluted CSF directly added to the wells.

### Kinetic calculations and statistical analysis

The assay cut-off was determined to be 52 h as a reproducible endpoint before the spontaneous amyloid aggregation in the wells with the tau KO mouse brain homogenate. The endpoint of 52 h was used for any data points with ThT fluorescence values at or greater than 52 h. The following parameters were analyzed: *F*_max_ (maximum ThT fluorescence), lag time (reaction time to exceed a ThT fluorescence threshold of the average baseline fluorescence + 5 standard deviations), time to reach maximum ThT fluorescence, and V_max_ (maximum slope). Differences in these parameters among the three groups with a dilution of 1 × 10^–1^ were assessed using One-way ANOVA with Bonferroni's Multiple Comparison Test. Statistical significance was defined as *p* < 0.05.

### Fluorescence resonance energy transfer (FRET) tau biosensor cell line

As described in Holmes et al. [36], the HEK293T cell line, engineered to express tau repeat domain (RD)-CFP and tau RD-YFP, both containing the P301S mutation, was kindly provided by Marc Diamond. The cells were cultured in T75 flasks using Dulbecco’s Modified Eagle Medium–high glucose (DMEM) (D6546, Sigma-Aldrich), supplemented with 10% Fetal Bovine Serum (FBS) (F7524, Sigma-Aldrich), 1% l-glutamine (G7513, Sigma-Aldrich), and 1% Penicillin/Streptomycin (P4333, Sigma-Aldrich), and passaged bi-weekly upon reaching 70–80% confluence.

For the transduction of tau seeds, cell lines were seeded at 70,000 cells per well in a 24-well plate. The next day, cells were transduced with the same brain homogenates as used for the RT-QuIC assay and τ306 substrate (control). Transduction complexes were made by combining 35 µL Opti-MEM (31985-047, Gibco) + 5 µL Lipofectamine 3000 (L3000-08, Invitrogen) with 16.8 µL Opti-MEM + 3.2 µL 3000 reagent (L3000-08, Invitrogen) + 20 µL brain homogenates for a total volume of 80 µL per well. Liposome preparations were incubated at room temperature for 20 min before adding to cells. After 48 h of incubation, cells were fixed with 4% PFA (P6148, Sigma-Aldrich) and imaged at 20× magnification with an Olympus BX63 scanning fluorescence microscope implementing the cellSens imaging software. Three representative images per sample were blindly evaluated using ImageJ to determine the percentage of cells with intracellular tau inclusions. Statistical analyses were performed using One-way ANOVA with Bonferroni's Multiple Comparison Test, with significance set at *p* < 0.05.

### Immunodepletion

Immunodepletion was performed using Pierce™ Protein A/G Magnetic Beads (88802, ThermoScientific) with AT8 (MN1020, Invitrogen) and tau5 (AB80579, Abcam) antibodies following the manufacturer’s protocol. Briefly, 25 µL of magnetic beads were resuspended in 500 µL of 1× Coupling Buffer. After collecting the beads with a magnetic rack and discarding the supernatant, 10 µg of AT8 or tau5 antibody was added to the beads, and the mixture was incubated for 15 min at room temperature. The beads were then collected, the supernatant removed, and the beads were washed three times with a coupling buffer. Next, 4 µL of 0.25 M Pierce disuccinimidyl suberate (DSS) (A39267, ThermoScientific) diluted in dimethyl sulfoxide (DMSO) (D2690, Sigma Aldrich) was added together with 50 µL of coupling buffer to crosslink the antibodies to the beads. This incubation was carried out for 30 min at room temperature. After collecting the beads and discarding the supernatant, 100 µL of elution buffer was added. This elution step was repeated twice, followed by washing the beads with ice-cold washing buffer. Next, 100 µL of brain homogenate from each group (Double-Negative, Intermediate, Double-Positive) was added to the beads crosslinked to either AT8 or tau5 antibodies, resulting in six samples in total. The samples were incubated overnight at 4 °C. The next day, the beads were collected, and the supernatants were retained for further analysis.

### Western blot

Immunodepleted (post-BH—AT8 and post-BH—tau5) and non-immunodepleted (pre-BH) samples from each group were analyzed by Western Blot using NuPAGE™ Bis–Tris Mini Protein Gels (NP0323BOX, Invitrogen). In total, 9 samples were mixed with 2-Mercaptoethanol (31350-010, Gibco) and LDS sample buffer (NP0007, Invitrogen), heated at 80 °C for 10 min, and loaded onto the gel. The gel was run in MOPS buffer (NP0001, Invitrogen) for 50 min at 180 V and transferred to a PVDF membrane (88520, ThermoScientific) at 100 V for 90 min. The membrane was blocked with 5% milk for 1 h and incubated overnight with pan tau antibody (rabbit, 1:5000) (A0024, Dako). Next day, the membrane wash was washed, incubated with HRP-conjugated secondary antibody (rabbit, 1:5000) (HAF008, RD systems) for 1 h, washed again, and visualized using Clarity and Clarity Max ECL Western Blotting Substrates (1705061, BioRad). The membrane was striped (46430, ThermoScientific), re-blocked, and incubated with GAPDH antibody (14C10; rabbit, 1:700) (2118S, Cell Signalling) as a loading control, followed by the same secondary antibody and visualization. ImageJ was used to quantify band intensities. Data were normalized to GAPDH and expressed as fold change over the control (pre-BH). Statistical analysis was performed using two-way ANOVA with the Dunnett Multiple Comparison Test.

### Quantification of astrocytes

To quantify reactive astrocytes, we performed IHC using an anti-GFAP antibody (rabbit, 1:1000) (2033429-2, Dako) and ImmPRESS (peroxidase) polymer horse anti-rabbit IgG reagent (MP-7401, Vector Labs). The neuropathological analysis of GFAP-IHC was carried out using StereoInvestigator software. Regions of interest of neocortex and white matter were outlined, and stained GFAP positive astrocytes were detected in the regions of interest with the automated “outline objects” function using manual adjustment of the sensitivity to accurately detect objects by an experimenter blind to experimental condition. Regions of interest and outlined astrocytes were saved and imported into Neurolucida explorer software (MBF Bioscience, Williston, VT USA) which was used to calculate the total area and area of GFAP staining for each region. GFAP burden was calculated with the formula: $\frac{\text{area occupied by GFAP staining in region of interest} }{\text{total area of region of interest}} \times 100$

The statistical analysis was performed using a One-way ANOVA test (Bonferroni), maintaining *p* < 0.05 as the criterion for statistical significance.

### Nuclei isolation and snRNA-seq

Nuclei were isolated as previously described [41] (refer to [27] for a detailed protocol). Briefly, frozen tissue was homogenized in 500 µL of lysis buffer (Nuclei Pure prep; NUC201-1KT, Sigma) using a disposable Eppendorf tube and incubated on ice for 12 min. The lysate was filtered through a 70 µm filter to remove debris and centrifuged at 500*g* for 5 min at 4 °C to pellet nuclei. The supernatant was discarded, and nuclei were resuspended in nuclei wash buffer, then filtered through a 40 µm filter. After another centrifugation at 500*g* for 5 min, the pelleted nuclei were resuspended in 250 µL of nuclei-wash buffer mixed with 250 µL of 50% iodixanol. This mixture was layered onto 500 µL of 29% iodixanol and centrifuged at 5000*g* for 20 min at 4 °C. For cell counting, 2 µL of acridine orange was added to 18 µL of the sample, analyzed using a Luna Countess to count nuclei, and the suspension was adjusted to the desired concentration for further experiments.

Then, snRNA-seq data was generated using the 10X Single Cell Next GEM Chip targeting a minimum of 5000 nuclei per sample and libraries prepared using the Chromium Single Cell 3′ Library and Gel Bead v3 kit according to the manufacturer’s instructions. cDNA libraries were sequenced using the Illumina NovaSeq 6000 system at a minimum sequencing depth of 30,000 paired-end reads per nucleus. All samples were sequenced on a single flow cell to avoid sequencing batch effects. All samples passed all internal QC steps. Raw base calls were converted to FASTQ files (Phred + 33 Illumina 1.9), a text-based format used to store biological data along with their quality scores and identification numbers. Adapter sequences, which are sequences ligated to the ends of cDNA fragments that can be introduced at the library preparation, were not detected. Therefore, the adapter trimming step was not needed (refer to Supplementary Fig. 2 for an overview of pre-processing steps and downstream analysis).

### Quality control of single-nucleus data

In this study, the analysis of FASTQ files commenced with the utilization of Cell Ranger software (version 3.1.0). Reads were mapped against the human reference genome GRCh38, encompassing intronic and exonic regions. Gene annotation was performed using Ensembl (version 93), culminating in generating raw and filtered cell matrices.

Ambient RNA-containing droplets, commonly called empty droplets, were discerned from droplets harboring authentic nuclei by implementing the CellBender algorithm [29]. The following parameters were applied: (i) false positive rate (FPR) set to 0.01; (ii) expected cells, denoting the anticipated number of nuclei from each sample, set to 10,000; (iii) total droplets included, representing the total number of droplets from the rank-ordered unique molecular identifier (UMI) plot, set to 15,000. The resultant output, a novel.h5 count matrix devoid of ambient RNA, was input for subsequent analyses.

Quality control procedures were conducted using the Seurat pipeline in the R statistical environment [14]. Briefly, for each sample, a Seurat object was instantiated utilizing the *CreateSeuratObject()* function. Genes detected in a minimum of 5 nuclei and nuclei exhibiting less than 10% mitochondrial reads, along with a minimum of 200 detected genes, were retained. Potential doublets were identified and removed using the DoubletFinder software [50], designed for predicting technical artifacts or instances where two or more nuclei appear as one in snRNA-seq data.

Default parameters were employed, including (i) the number of statistically significant principal components (PCs) set to 10; (ii) the number of generated artificial doublets (pN) expressed as a proportion of the merged real-artificial data set to 0.25; (iii) a parameter (pK) used to compute the proportion of artificial nearest neighbors set to 0.09; (iv) the total number of predicted doublets (nExp), estimated from cell density loads used in the 10 × device, varied per sample. DoubletFinder simulated artificial doublets by calculating the average transcriptional profile of randomly selected nuclei pairs and subsequently identified doublets by comparing the profiles of these artificial doublets to "singlets" nuclei. Nuclei with a high proportion of artificial neighbors, determined by dividing the number of artificial neighbors by the neighborhood size in gene expression space, were excluded from the dataset.

### Integration of samples and cell-type identification

Following the removal of doublets, an integrated data matrix was constructed to facilitate subsequent analyses. Integration of individual samples was executed using the Seurat integration pipeline. Initially, each dataset underwent normalization for sequencing depth employing SCTransform [34], a tool within the Seurat framework that applies variance stabilization and transformation. SCTransform mitigates technical variations, particularly sequencing depth (total unique molecular identifier (UMI) counts per cell), by modelling counts through a regularized negative binomial model. Variance adjustment is achieved by amalgamating information across genes with similar abundances post-transformation. The rationale for variance adjustment lies in the observation that in single-cell RNA sequencing (scRNA-seq) data, cells with low UMI counts exhibit disproportionately higher variance for highly abundant genes, thereby reducing the contribution of variance from other lowly abundant genes. Briefly, before integration, the *SCTransform()* function with default parameters was employed to normalize each sample. The *SelectIntegrationFeatures()* function was utilized to choose 3000 features for integration. Subsequently, the *PrepSCTIntegration()* function, followed by the *FindIntegrationAnchors()* function, identified anchors representing cell pairwise correspondences between single cells. Finally, the *IntegrateData()* function was applied to generate an integrated assay dataset comprising: (i) all UMI counts; and (ii) centered and corrected Pearson residuals. Visualization of the integrated assay dataset was achieved through Uniform Manifold Approximation and Projection (UMAP) post-integration. Seurat functions, namely *FindNeighbors()* and *FindClusters(),* were employed to compute nearest neighbors and identify clusters using a shared nearest neighbor modularity optimization based on the Louvain clustering algorithm. The *FindAllMarkers()* function was subsequently employed to identify marker genes for each cluster, representing differentially expressed genes (DEGs) in each cluster compared to all other clusters. To assign cell types to each cluster, Fisher's exact test was utilized to examine the overlap between a cluster and a list of cell-type markers obtained from Wang et al. [66].

### Differential gene expression

We applied MAST [28] for each pair-wise comparison to identify genes that were differentially expressed across the three groups (after adjusting for sex, age, and individual ID). Across all pairwise comparisons, DEGs were identified based on a false discovery rate (FDR) threshold of 0.05 and a log2 fold change greater than log2(1.5).

### Functional enrichment of DEGs

Once DEGs were identified for each pair-wise comparison in each cell-type, WEB-based GEne SeT AnaLysis Toolkit (WebGestaltR) [47] (v 0.4.4) was employed to extract underlying biological themes from the identified gene sets. Default parameters were applied, including a minimum gene overlap of 10, a maximum of 500 genes, and multiple testing corrections using the Benjamini-Hochberg (BH) method. Significant pathways were identified using an FDR threshold of 0.05. Gene sets were divided into up- and down-regulated DEGs across the main cell types, resulting in 14 gene sets per pairwise comparison. Each set was tested for enrichment against gene sets annotated with Biological Process (BP) Gene Ontology (GO) terms.

For further analysis, the R package rrvgo [61] was used to reduce redundancy in the enriched GO terms. FDR scores for each GO term were extracted from the WebGestaltR results and assigned to their corresponding GO term identifiers. A similarity matrix was calculated using the "Wang" method based on BP GO terms, constructed with the *calculateSimMatrix* () function using the org.Hs.eg.db database for human gene annotation [18]. To reduce redundancy within the similarity matrix, the *reduceSimMatrix*() function was applied with a similarity threshold of 0.7. The reduced terms, representing non-redundant GO parent terms, were extracted and visualized using the R package ggplot2 [69].

### Enrichment of heritability analysis

LDSC [1, 31], was used to prioritize DEG sets associated with neurological phenotypes based on publicly available AD GWAS summary statistics [37]. LDSC is a technique widely used to estimate heritability. LDSC estimates heritability by considering that SNPs in higher LD with other SNPs have higher test statistics on average for a polygenic trait/disease because more causal SNPs being tagged. In other words, LDSC quantifies the contributions of polygenic effects and estimates its bias by assessing the relationship between LD scores and test statistics of SNPs from GWASs. The analysis was carried out as follows (after converting GWAS summary stats to LDSC format using (munge sumstats.py)): (i) annotation files were generated for each gene set using default parameters. SNPs were then mapped to genes using dsSNP file NCBI Build 37 co-ordinates (build 147 and hg19); (ii) LD scores were computed for each annotation file using a 1 cM window (restricted to Hapmap3 SNPs), and the full baseline model was downloaded from the LDSC GitHub page (https://github.com/bulik/ldsc); (iii) an enrichment score and its corresponding p-value were calculated based on the proportion of total SNPs per annotation, after taking into account all other annotations. Annotation categories with significant positive enrichment of SNP-heritability (tested using a one-tailed test) are then reported as a final result.

### Single-nucleus trajectory

To address the issue of cellular asynchrony, a significant factor contributing to the observed high variability in gene expression across snRNA-seq data, we employed Monocle [17]. This tool was utilized to construct trajectories, enabling the identification of changes in pseudo-temporal trajectories within the distinct groups under investigation. Monocle employs a semi-supervised approach to order individual cells or nuclei along a trajectory, specifically aligning with the tau oligomers load in our analysis. Notably, nuclei from the Double-Negative group served as the baseline reference, and Monocle assigned pseudotime values to each remaining nucleus, signifying its position along the specified trajectory. This approach facilitated the comprehensive exploration of dynamic changes in gene expression patterns, mitigating the impact of cellular asynchrony on the interpretation of the snRNA-seq data.

### Code and data availability

The raw and processed sequencing data generated in this study have not yet been deposited. All scripts used in this study can be found in GitHub Supplementary materials including full tables https://github.com/rahfel/tauPLA.

## Results

### Tau-PLA reveals extensive tau multimerization in temporal and occipital cortices in early AD Braak stages

**Fig. 1:**
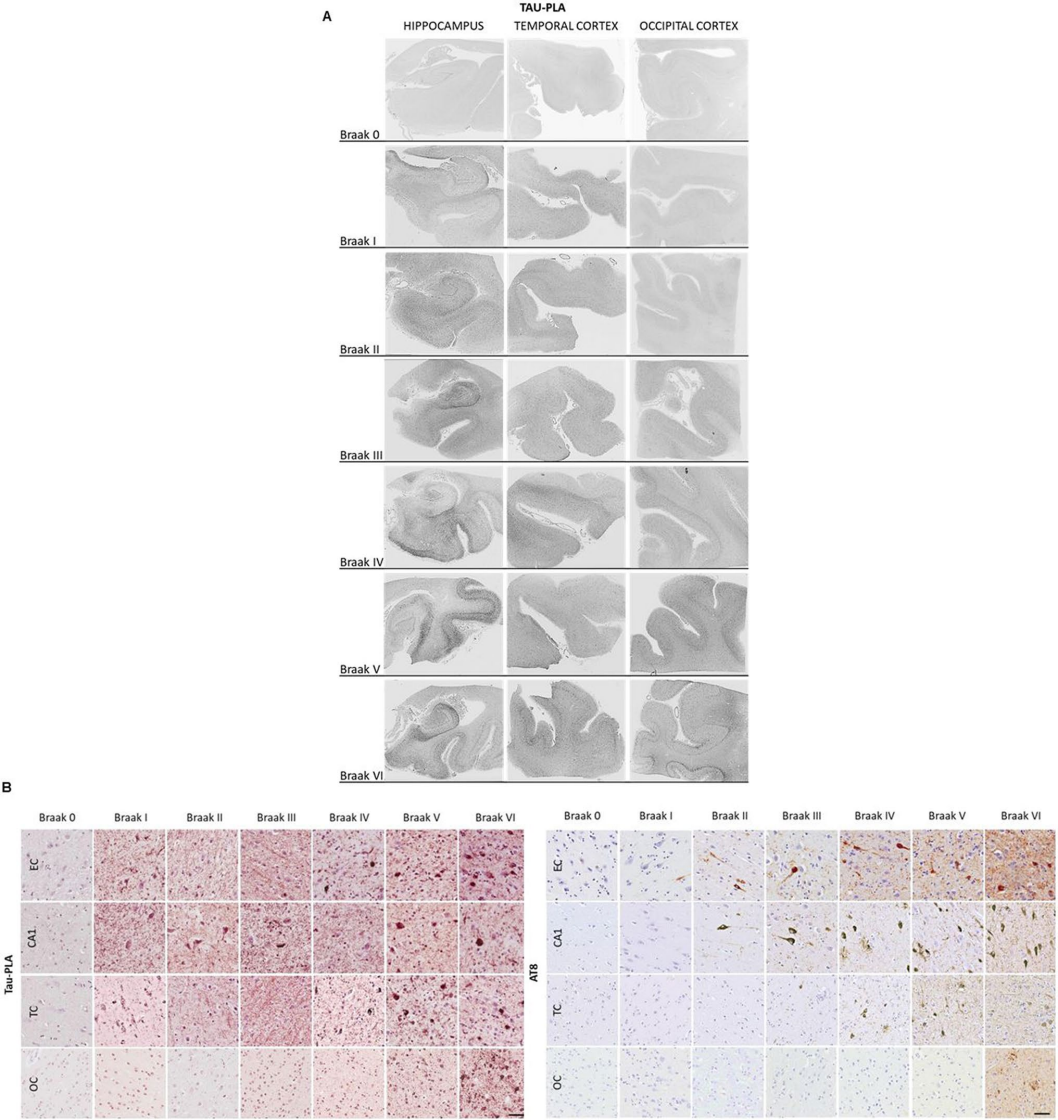
Early tau multimerization detection across the different Braak Stages as defined by BrainNet Europe diagnostic protocol. **A** Scanned tau-PLA labeled post-mortem human brain tissue sections. Sections from the hippocampal region, temporal cortex (MTC), and occipital cortex were stained for tau-PLA. **B** FFPE sections from postmortem human brain tissue from the brain regions of the hippocampus, temporal (MTC), and occipital cortices were stained for AT8-IHC and tau-PLA. The Braak stage of each case was determined according to the modified Braak staging system based on AT8-immunoreactive NTs across the brain tissue. Scale bar 50 μm. *EC e*ntorhinal cortex, *CA1* hippocampus, *TC* temporal cortex, *OC* occipital cortex, *MTC* middle temporal cortex

Building upon our prior research analyzing the spatial and temporal distribution of tau-tau interactions in hippocampal regions [9], this study expanded to include temporal and occipital cortices, which are regions affected along the Braak tau pathway following the hippocampal region. For this purpose, we employed tau-PLA alongside IHC on human post-mortem tissue samples spanning all neurofibrillary Braak stages from 0 to VI (Supplementary Table 1a). Consistent with our previous findings [9], tau-PLA labeled both large perikaryal inclusions (mostly NFTs) and a diffuse cellular and neuropil pattern (Fig. 1A, B). Automated quantification of the amount of pathology within these two patterns showed that labeling of perikaryal lesions by tau-PLA paralleled that obtained with AT8-IHC in all regions (Supplementary Fig. 3). In contrast, diffuse tau-PLA signal was present (defined as significantly higher than in Braak 0 stage) from Braak stage I in the hippocampal regions and the inferior temporal cortex, from Braak stage II in middle and superior temporal cortices, from Braak stage III in parastriate occipital cortex, and from Braak stage IV in the striate occipital cortex (Fig 1B, and Supplementary Fig. 3b). This signal preceded the expected AT8 signal for these regions and stages based on the literature [13] and our own AT8 perikaryal lesion quantification in this cohort, where significant AT8-IHC stained perikaryal lesions were present in temporal cortices from Braak stage IV and in the occipital striate cortex in stage VI (Supplementary Fig. 3a).

To further dissect the evolution of tau molecular events, we utilized tau-PLA in combination with IHC using a panel of antibodies targeting phosphorylated and misfolded tau (AT8, AT180, pT217, PHF1, pS422, Alz50, and MC-1). While both PLA-labeled perikaryal lesions and diffuse signal are easily quantified by automated methods given the morphological regularity of the PLA dots, smaller structures labeled by IHC may be missing. Therefore, and as previously described [9], to evaluate the tau antibody IHC panel alongside tau-PLA, we separately quantified small-sized stained structures (the diffuse pathology recognized by tau-PLA and dot-/thread-like labeling by tau IHC) and larger perikaryal NFT-like structures. A blind semi-quantitative evaluation of small-sized stained structures labeled by tau-PLA and IHC for tau antibodies was performed using reference scales (Supplementary Fig. 1a), while automated quantification was used for the larger perikaryal lesions.

**Fig. 2:**
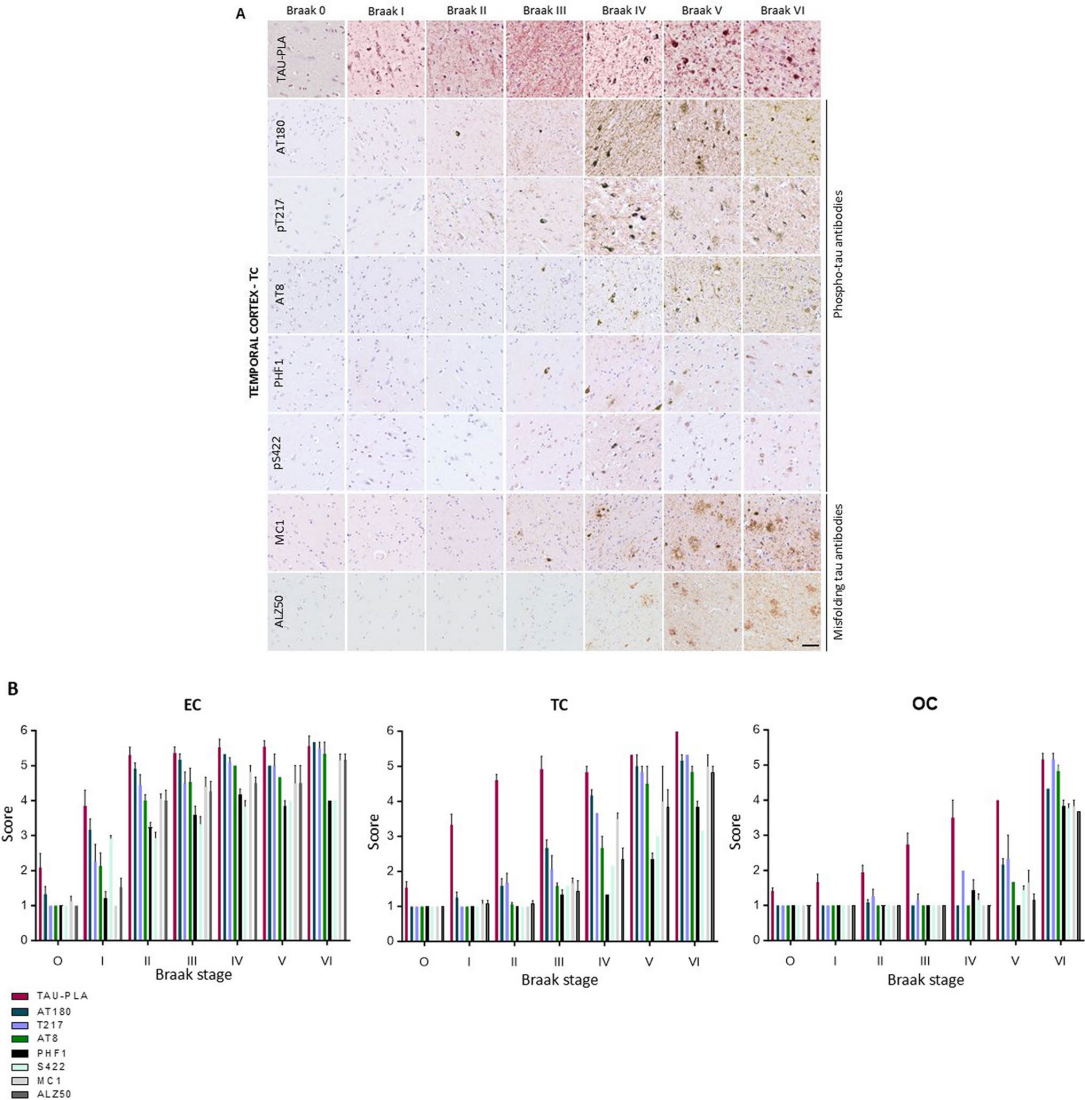
Diffused tau pathology labeled with different phospho-tau and conformational tau antibodies across the different Braak stages as defined by BrainNet Europe diagnostic protocol. **A** FFPE sections from post-mortem human brain tissue from the middle temporal cortex stained for AT180-, T217-, AT8-, PHF1-, S422-, MC1-, and Alz50-IHC. Scale bar 50 μm. **B** Quantification of diffuse tau pathology labeled with tau-PLA and various phospho-tau and conformational tau antibodies across Braak stages in EC, TC, and OC. The *y*-axis represents a semi-quantitative score from 0 to 6 (Supplementary Fig. 1a), while the *x*-axis corresponds to Braak stages (B0–BVI). Each antibody at each Braak stage was compared to Braak 0 through a One-way ANOVA (Dunnett); *N* = 11/12/12/9/7/8/8. Each bar represents the mean ± standard error of the mean (SEM). Full statistical details can be found in Supplementary Table 2. *EC* entorhinal cortex, *TC* temporal cortex, *OC* occipital cortex

In agreement with our automated quantification of diffuse tau-PLA signal, we found that overall, tau-PLA recognized multimers from an earlier stage than any of the other markers in most brain regions (Fig. 2A, B). Using semi-quantitative scales, the levels of tau multimers detected by tau-PLA showed statistically significant differences when compared to Braak stage 0. Specifically, differences were observed starting from Braak stage I in the entorhinal and temporal cortices, and from Braak stage III in the occipital cortex (Fig. 2A, B, and Supplementary Table 2). AT180-IHC revealed small tau structures as early as Braak stage II in the entorhinal cortex, from stage III in the temporal cortex, and from stage V in the occipital cortex. T217-IHC detected small-sized tau-stained structures beginning at Braak stage II in both the entorhinal and temporal cortices and at Braak stage IV in the occipital cortex. AT8-IHC identified small tau structures from Braak stage I in the entorhinal cortex, from stage III in the temporal cortex and from stage V in the occipital cortex. Other phospho-tau antibodies and conformational markers were less effective in capturing small-sized diffuse pathology (refer to Fig. 2A, B, and Supplementary Table 2 for detailed quantifications). Regarding the detection of larger, perikaryal, inclusion-type lesions, AT180 and T217 antibodies also appeared to be the most sensitive (Supplementary Fig. 4). These results further support the idea that tau multimerization is an early, critical event in the progression of AD-type tau pathology, detectable even before enough tau phosphorylation and misfolding become detectable at the tissue level via IHC, and before widespread tau inclusion pathology develops along the Braak tau pathway. In addition, it identified the temporal cortex as a valid region for studying early tau multimeric pathology in the absence of significant NFTs and for comparing it with that associated with incipient NFT load.

### Characterization of samples for seeding amplification assays and transcriptomic analyses

**Fig. 3:**
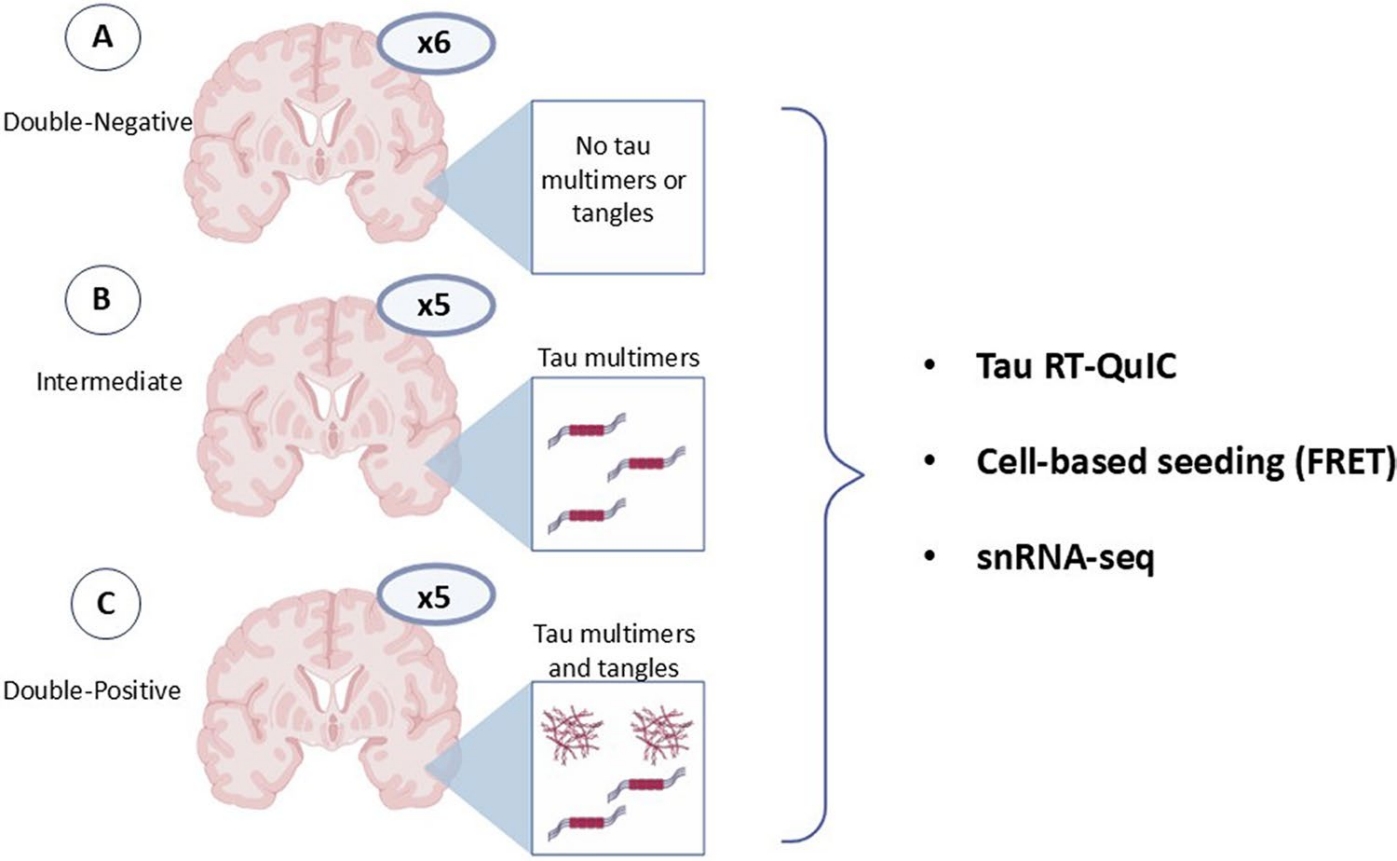
Overview of samples used for tau RT-QuIC, cell-based seeding (FRET), and snRNA-seq assays. 6 subjects were designated to the Double-Negative group, exhibiting no detectable tau aggregates, implying an absence of tau pathology (**A**); 5 subjects were designated to the Intermediate group, demonstrating early signs of tau multimerization with a minimal or negligible number of large fibrils (**B**); 5 subjects were designated to the Double-Positive group, characterized by both diffuse tau pathology and pronounced large lesions in the temporal region (**C**). *RT-QuIC* Real-Time Quaking-Induced Conversion, *FRET* fluorescence resonance energy transfer, *SnRNA-seq* single-nucleus RNA sequencing

To further explore the functional and transcriptional changes in the early stages of AD-type tau pathology, we focused on an in-depth analysis of the middle temporal cortex, ideally situated halfway through the Braak tau pathway. This region allowed us to capture both early tau multimerization and incipient NFT formation in preclinical disease. As described in the material and methods, we selected cases with available frozen tissue and categorized them into three groups based on tau-PLA and AT8-IHC staining to capture the spectrum of tau pathology (Fig. 3). The first group, designated Double-Negative (tau-PLA−/AT8−; *N* = 6), exhibited no significant tau aggregates. The second group, labeled Intermediate (tau-PLA+/AT8−; *N* = 5), demonstrated early signs of tau multimerization within the cortex, as detected by tau-PLA, but no significant AT8-IHC signal. A threshold of > 30 tau-PLA diffuse particles was set to categorize a sample as tau-PLA positive. This cut-off, corresponding to the upper limit of the 99% confidence interval for the tau-PLA number of particles in Braak 0 samples, was calculated using the formula:

$$CI = \overline{x} \pm t\left( {\sigma /\sqrt n } \right)$$

*x̄* represents the mean tau-PLA particle count in Braak 0 cases, *t* is the *t*-value of the *t*-distribution for the degrees of freedom (number of Braak 0 cases minus 1) at a 99% confidence level, *σ* is the standard deviation of tau-PLA particle counts in Braak 0 cases, and *n* is the number of Braak 0 cases. A cut-off of > 1 particle was set to define AT8-positive samples. This threshold, corresponding to the upper limit of the 99% confidence interval for the AT8 particle count in Braak 0 samples, was determined using the same formula, calculated in the same manner as described above:

$$CI = \overline{x} \pm t\left( {\sigma /\sqrt n } \right)$$

Specifically, 1 particle (rounded up to the nearest whole number) represented this upper limit. The third group, labeled Double-Positive (tau-PLA+/AT8+; *N* = 5), was characterized by both diffuse tau pathology and pronounced large lesions in the temporal region, signifying a more advanced stage of tau AD-type tau pathology. A more detailed quantitative and comparative analysis of the AT8-IHC and tau-PLA pathologies across these three groups is presented in Supplementary Fig. 1b and Supplementary Table 1b.

To further characterize distinct tau species across the groups and to aid in the interpretation of the seeding amplification assays, western blot analysis of brain homogenates immunodepleted with AT8 or tau5 antibodies was performed (Supplementary Fig. 5). In both the Double-Negative and Intermediate groups, AT8 immunodepletion did not statistically significantly affect total tau levels, while tau5 immunodepletion did, suggesting that the bulk of total tau in these groups was made up of non-AT8-phosphorylated tau. In the Double-Positive group, immunodepletion with both AT8 and tau5 antibodies markedly reduced total tau levels, indicating that in this group, which has evident NFT formation, AT8-phosphorylated tau made up a significant component of total tau.

### Tau RT-QuIC reveals high seeding activity of tissue bearing early tau multimers

To investigate the seeding activity of early tau multimers detected by tau-PLA, we performed tau RT-QuIC using the τ306 substrate with a point mutation at cysteine 322 to serine. The τ306 fragment, encompassing tau residues 306–378 of the full-length 4R tau isoform and located in the core of tau fibrils, was utilized (refer to Supplementary Fig. 6). In the initial stage, we performed tau RT-QuIC with soluble τ306 fragment or τ306 aggregates, with or without the addition of tau KO mouse brain homogenate and N-2 supplement, to investigate assay conditions (refer to Supplementary Fig. 6b). The results demonstrated the essential role of tau KO mouse brain homogenate and N-2 supplement in preventing the spontaneous aggregation of τ306 monomeric substrate. Furthermore, the addition of τ306 aggregated species to the tau RT-QuIC reaction solution induced rapid seeding activity of the tau-based monomeric substrate, resulting in the prompt formation of τ306 aggregates (refer to Supplementary Fig. 6b). Prior to conducting the assay, both soluble and aggregated τ306 species were examined using TEM (refer to Supplementary Fig. 6c). T306 aggregates were generated through the agitation of recombinant τ306 fragment in the presence of heparin.

**Fig. 4:**
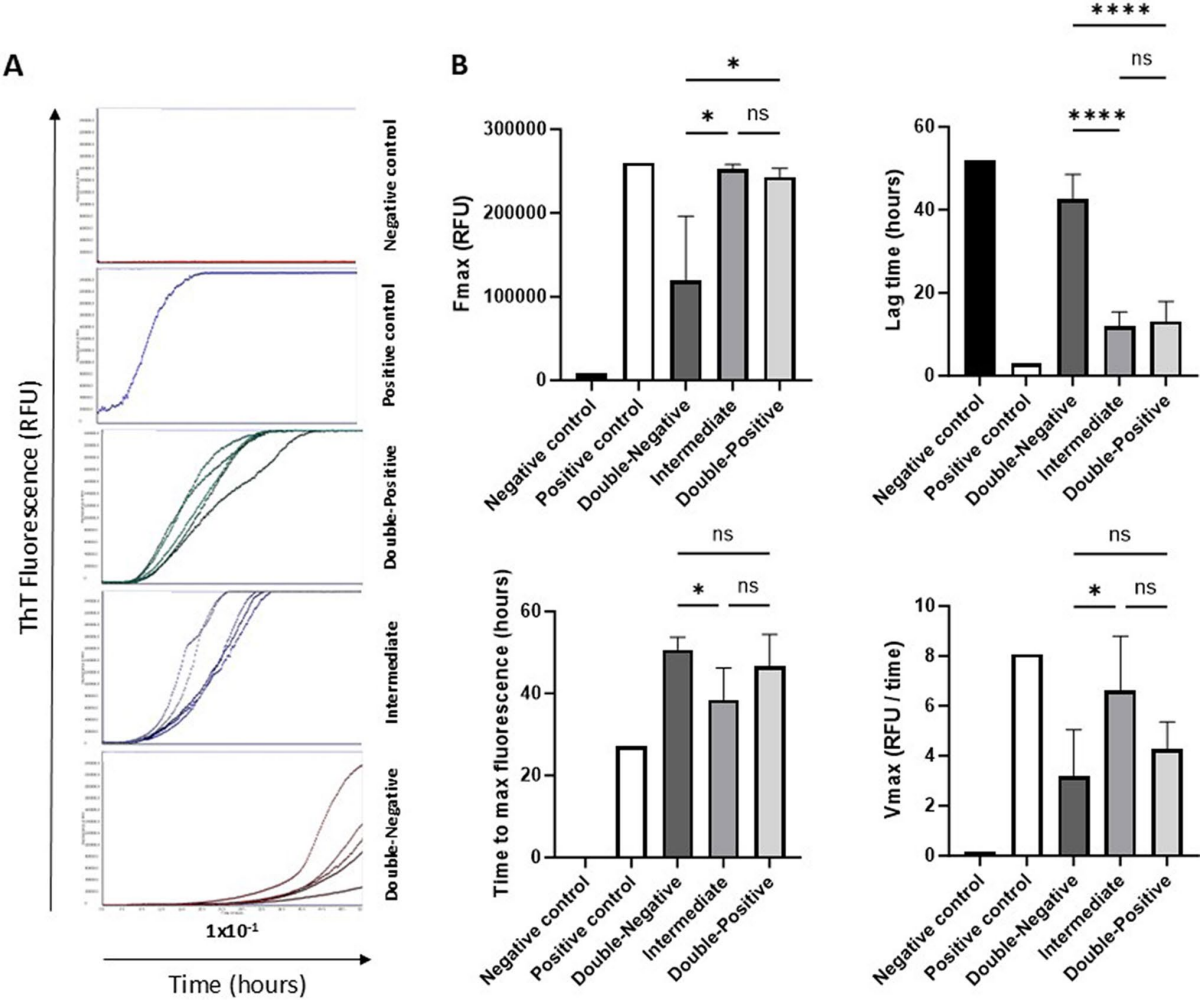
Tau RT-QuIC analysis. **A** RT-QuIC analysis of negative control (tau MAPT KO mouse brain homogenate), positive control (τ306 aggregates), Double-Negative, Intermediate, and Double-Positive brain homogenates. Each curve represents a single case, run in triplicate. **B** Comparison of tau seeding activity of negative control (tau MAPT KO mouse brain homogenate), positive control (τ306 aggregates), Double-Negative, Intermediate, and Double-Positive brain homogenates with RT-QuIC. *F*_max_ (maximum ThT fluorescence), lag time (reaction time to exceed a ThT fluorescence threshold of the average baseline fluorescence + 5 SD), time to reach maximum ThT fluorescence, and V_max_ (maximum slope) were analyzed. The assay cut-off was determined to be 52 h as a reproducible endpoint before the spontaneous amyloid aggregation in the negative control wells. The endpoint of 52 h was used for any data points with ThT fluorescence values at or greater than 52 h. Groups with dilution 1 × 10^–1^ were assessed through a One-way ANOVA (Bonferroni); *N* = 6/5/5. Each bar represents the mean ± standard deviation (SD). *p < 0.05, **p < 0.01, ***p < 0.001, ****p < 0.0001

Following the assessment of assay conditions, we conducted a tau RT-QuIC dilution analysis of brain homogenates obtained from the temporal region of Double-Negative, Intermediate, and Double-Positive cases. T306 aggregates and tau KO mouse brain homogenates served as positive and negative controls, respectively. Brain homogenates from the Double-Negative group exhibited slower seeding activity compared to the Intermediate and Double-Positive groups, where the τ306 substrate displayed rapid seeding activity (Fig. 4A). The tau seeding activity among the groups was compared using several parameters, including the maximum ThT fluorescence (*F*_max_), the lag time (the time required to reach a ThT fluorescence value equal to the average baseline fluorescence + 5 SD), the time to reach the maximum ThT fluorescence, and the maximum slope (*V*_max_). The *F*_max_ values of the Intermediate and Double-Positive groups were significantly higher compared to the Double-Negative group, with the Intermediate group exhibiting the shortest time to reach the maximum ThT fluorescence (Fig. 4B). Lag time analysis revealed that the lag phase before reaching the ThT fluorescence threshold was significantly shorter in the tau RT-QuIC reactions seeded with Intermediate and Double-Positive brain homogenates compared to the Double-Negative brain tissue (Fig. 4B). Moreover, the V_max_ of the kinetic curve in the Intermediate group was significantly higher than that of the Double-Negative group, but there was no statistical difference between the Intermediate and Double-Positive groups (Fig. 4B). These data indicate higher seeding activity in the temporal brain tissue of the Intermediate and Double-Positive groups compared to the Double-Negative controls lacking any tau pathology. Although CSF samples from the Double-Positive group were unavailable, analysis of available CSF samples also demonstrated significantly higher seeding activity in the Intermediate group compared to the Double-Negative group (Supplementary Fig. 7).

### Validation of tau seeding activity using cell-based seeding assay

**Fig. 5:**
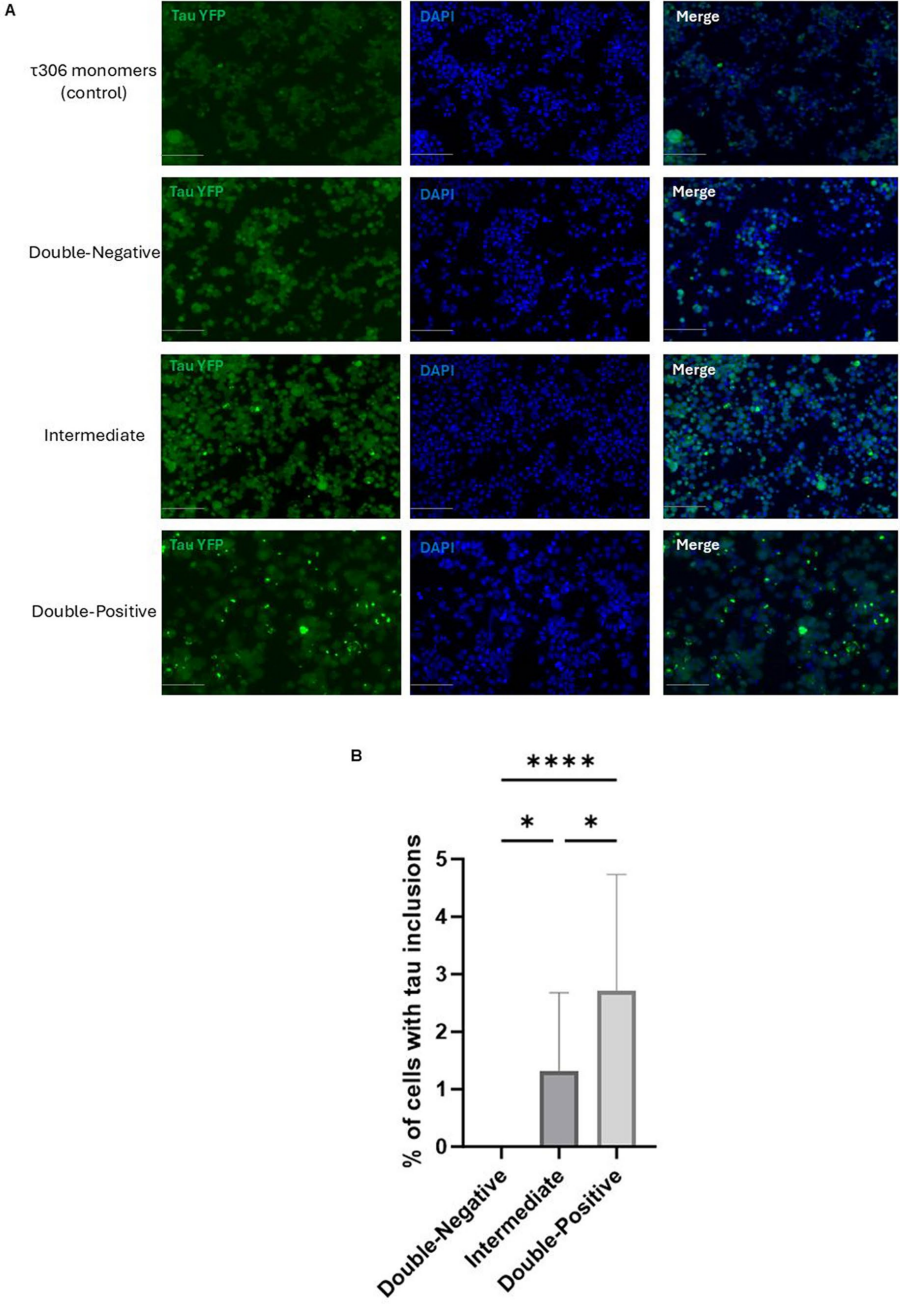
Seeding amplification assay using tau biosensor cell line. Comparison of tau seeding activity of Double-Negative, Intermediate, and Double-Positive brain homogenates of temporal with FRET-based biosensor cell line. τ306 monomers were used as a control. **A** Fluorescent imaging showcases cells post 48-h treatment with respective brain homogenates; scale bar = 50 µm. **B** Quantitative analysis displaying the percentage of cells with intracellular tau inclusions, assessed through one-way ANOVA (Bonferroni); *N* = 6/5/5. Each bar represents the mean ± standard deviation (SD). *p < 0.05, **p < 0.01, ***p < 0.001, ****p < 0.0001

We utilized an additional seeding amplification technique to corroborate the findings from the RT-QuIC seeding assay. This cell-based seeding assay exposed the HEK293T cell line, engineered to express tau proteins RD-CFP and RD-YFP with the P301S mutation, to the same brain homogenates used in the RT-QuIC assay. As expected, results highlighted in Fig. 5 showed that Double-Positive group exhibits significant numbers of intracellular tau inclusions, indicating the seeding activity of tau. Crucially, the Intermediate group also displayed substantial tau inclusions in vitro (Fig. 5), despite the absence of significant NFTs pathology in the post-mortem tissue. This finding is particularly significant as it underscores this assay's ability to detect early and potentially pre-symptomatic stages of tau aggregation. The absence of tau inclusions in the control samples (Double-Negative) further confirms the assay's specificity and sensitivity (Fig. 5).

### Validating the involvement of different tau species in seeding

**Fig. 6:**
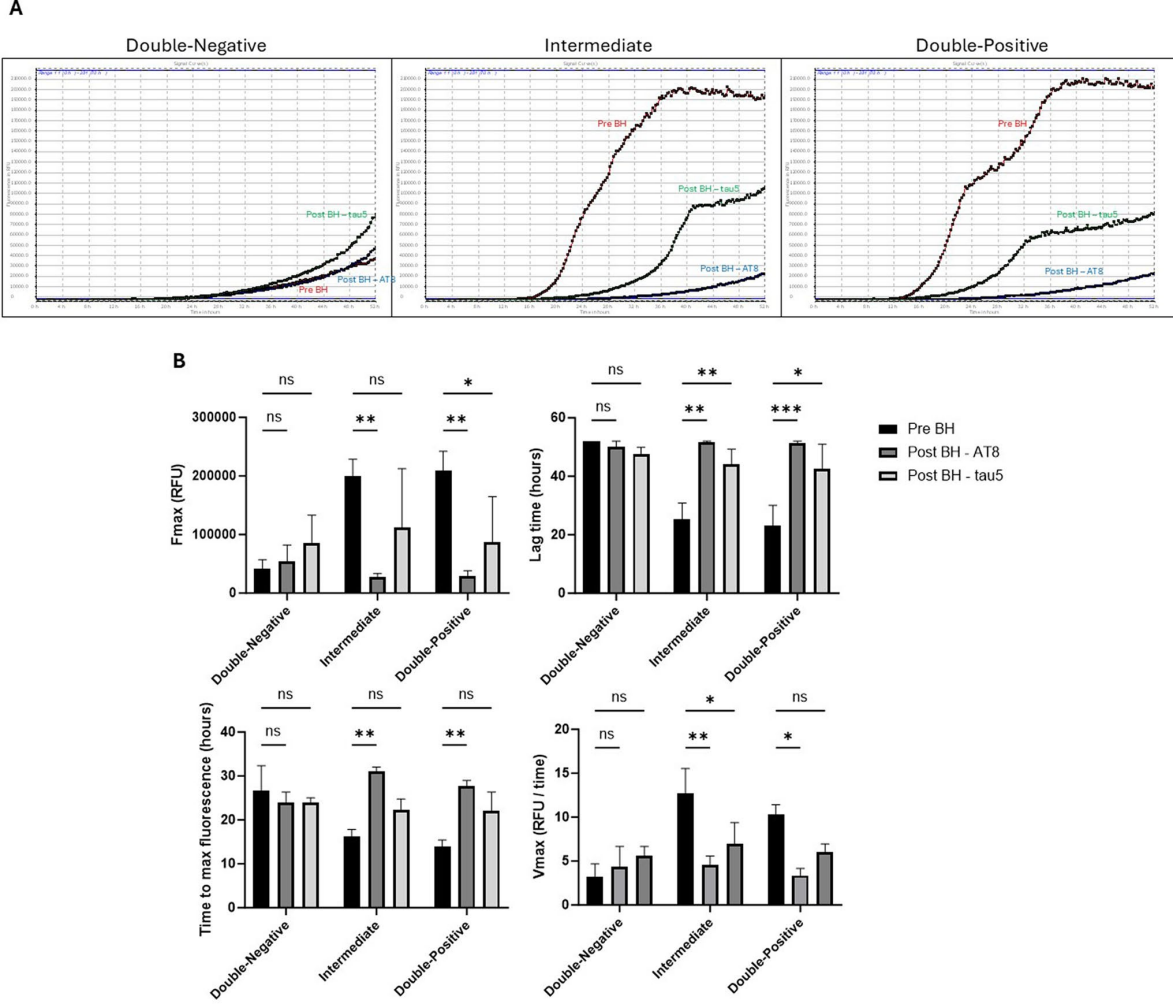
Seeding activity of non-immunodepleted and immunodepleted brain homogenates assessed by tau RT-QuIC. **A** Fluorescence curves of non-immunodepleted brain homogenates (pre-BH, visualized in red), immunodepleted brain homogenates with AT8 antibody (post-BH—AT8; visualized in blue), and immunodepleted brain homogenates with tau5 antibody (post-BH—tau5; visualized in green) across Double-Negative, Intermediate, and Double-Positive groups. **B** Comparative analysis of seeding kinetics, including maximum fluorescence (*F*_max_), lag time (reaction time to exceed a ThT fluorescence threshold of the average baseline fluorescence + 5 standard deviations), time to maximum fluorescence, and maximum aggregation rate (*V*_max_), revealing differences in tau aggregation potential between groups and immunodepletion conditions. One sample from each group was analyzed in triplicates. Data was evaluated using Two-way ANOVA comparing to the control (pre-BH) followed by Dunnett's Multiple Comparison Test. Each bar represents the mean ± standard error of the mean (SEM). *p < 0.05, **p < 0.01, ***p < 0.001

To assess the seeding activity of different tau species, tau-immunodepleted brain homogenates were tested by tau RT-QuIC (Fig. 6). In the Double-Negative group, all conditions, including pre-immunodepletion (pre-BH) and post-immunodepletion with AT8 and tau5 antibodies (post-BH—AT8 and post-BH—tau5), displayed minimal seeding activity, consistent with the absence of tau pathology (Fig. 6A). In contrast, the Intermediate and Double-Positive groups exhibited high seeding activity in the pre-BH samples, indicating the presence of seeding-competent tau species. Notably, immunodepletion with the AT8 antibody significantly reduced seeding activity in both groups, as reflected by significant decrease in maximum fluorescence (*F*_max_), significant increase in lag time, a delay in the time to reach maximum fluorescence, and a lower aggregation rate (*V*_max_) (Fig. 6B). Immunodepletion with tau5 significantly reduced, but did not completely abolish, seeding activity in both the Intermediate and Double-Positive groups, as indicated by the persistent fluorescence signals.

### Transcriptomic profiling of the temporal cortex

SnRNA-seq analysis was performed on the same cohort representing 16 subjects with no clinical history of AD (Fig. 3). Samples were matched for demographic factors as far as possible. However, there was a difference in the proportions of sexes between groups due to sample availability (refer to Supplementary Table 1b for sample demographics). Overall, quality measures for read mapping and sequencing were high (Supplementary Table 3). In total, snRNA-seq was generated on 52,008 nuclei. Across all samples, 17,483 genes were expressed (defined as genes with > 1 reads detected in at least 5 nuclei), with an average of 1923 unique genes detected per nucleus, defined as genes with > 0 reads detected in at least 5 nuclei (refer to Supplementary Table 3 for full metrics).

### Cell-type identification

The snRNA-seq datasets were integrated using the R-package *Seurat* [14]. An integrated plot was generated to visualize clusters representing the major brain cell types (Supplementary Fig. 8). Clusters were assigned to cell type based on enrichment of cell-type markers derived from Wang et al. [67]. In total, there were 18,492 nuclei identified as excitatory neurons, 4823 as astrocytes, 3670 as inhibitory neurons, 18,267 as oligodendrocytes, 2535 as oligodendrocyte precursor cells (OPC), 3671 as microglia, and 550 as vascular (refer to Supplementary Table 4a and Supplementary Table 4b).

### Differential expression analysis reveals consistent expression patterns in Intermediate and Double-Positive groups

**Fig. 7:**
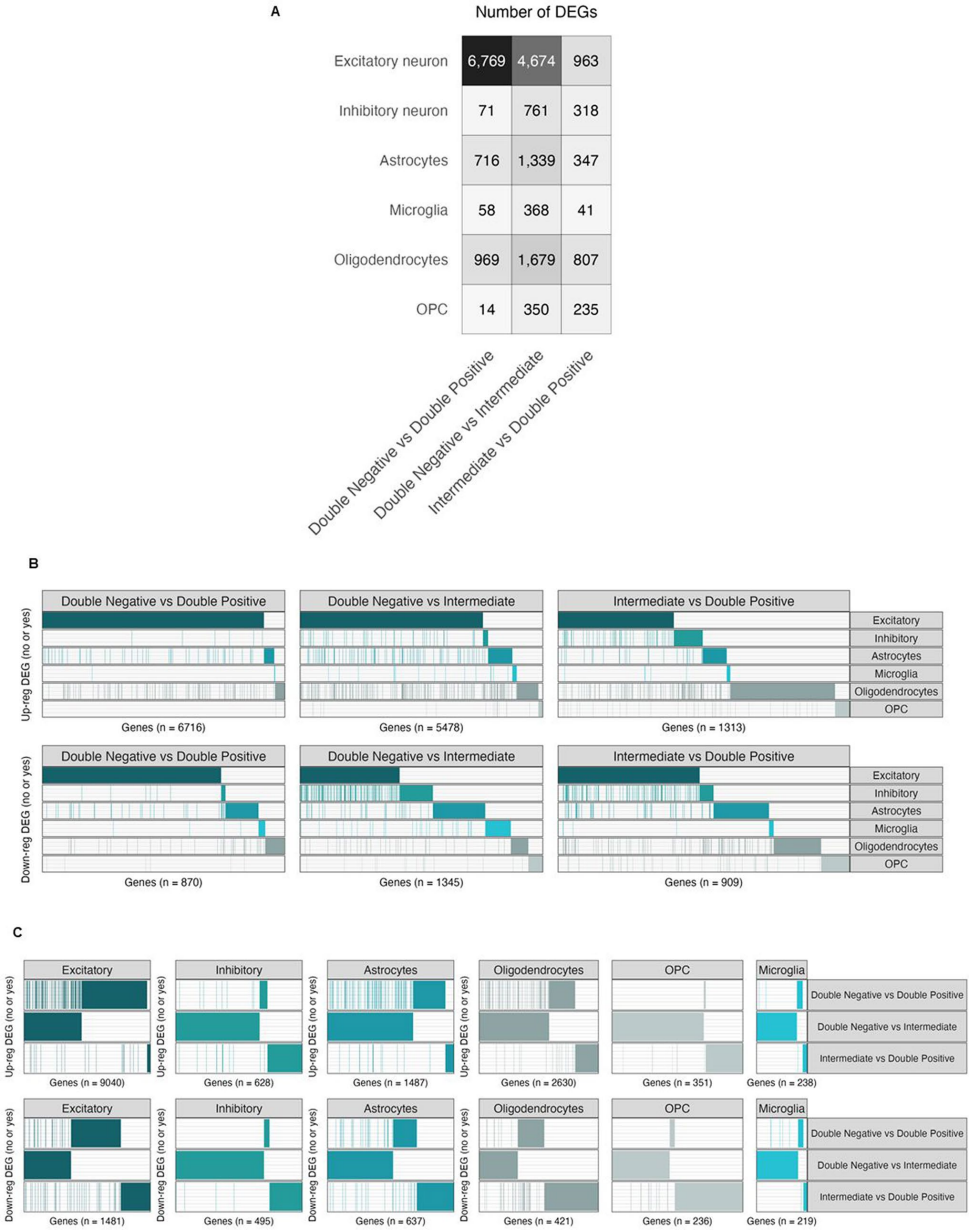
Cell-type-specific gene expression changes across all comparisons. **A** Total number of DEGs across all pairwise comparisons (FDR < 0.05, log2(fold change) > log2(1*.*5)). **B**, **C** A yes/no plot indicating if a gene (column) is expressed in a given comparison. The total number of DEGs (unique) in each comparison is indicated on the *x*-axis. The full list of DEGs across all pairwise comparisons in the panel is available in Supplementary Table 5

**Fig. 8:**
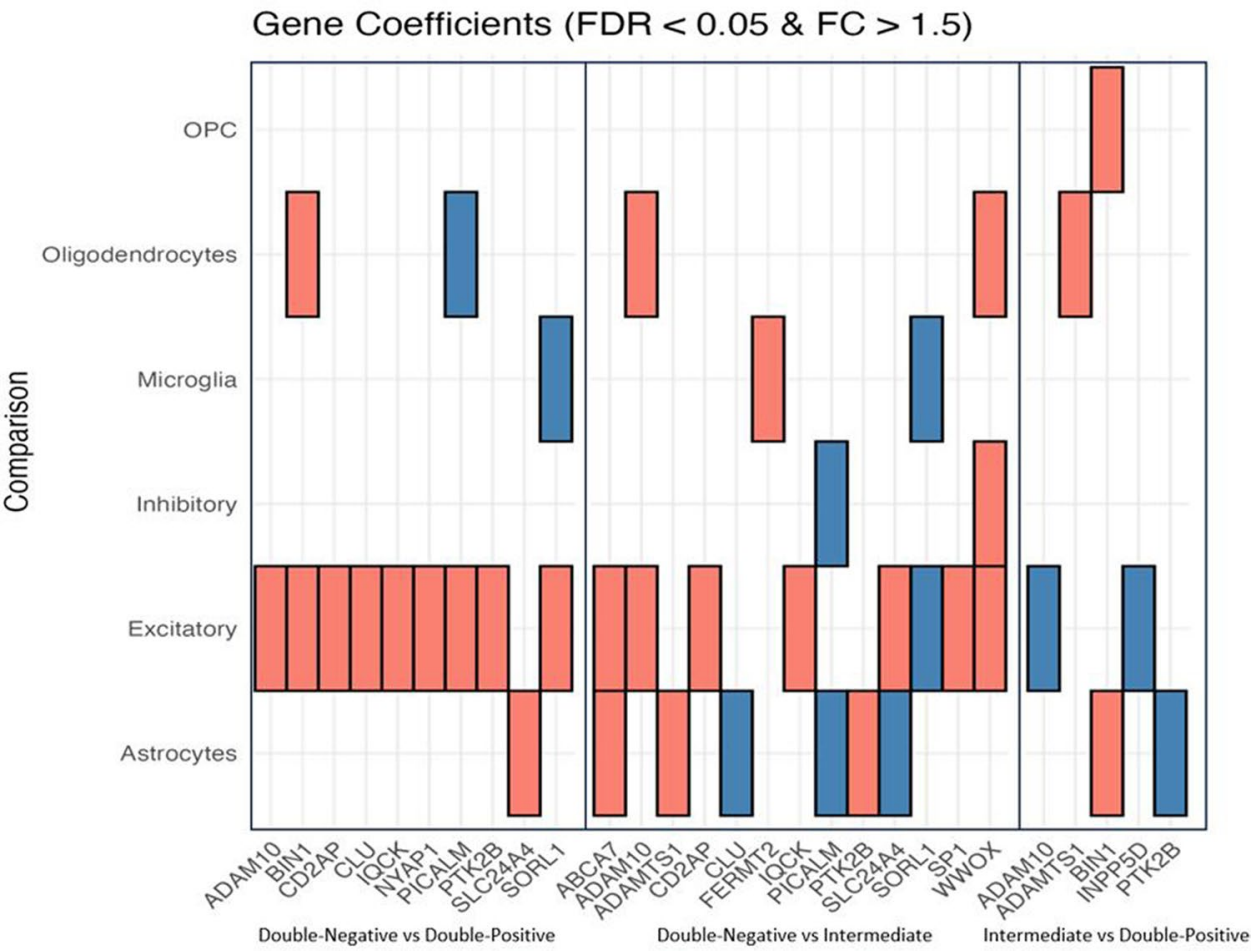
Expression levels of previously identified Alzheimer’s Disease-relevant genes in the current datasets. Red color denotes up-regulated genes, while blue color denotes down-regulated

To investigate transcriptional changes specific to cell types across the three experimental groups, we conducted differential expression analysis (DEA) for six major cell types in pairwise comparisons (Fig. 7). The analysis revealed pronounced differences between the Double-Negative and Double-Positive groups, with fewer changes observed in comparisons involving the Intermediate and Double-Positive group. In excitatory neurons, a striking 6128 genes were up-regulated and 641 down-regulated in Double-Positive samples compared to Double-Negative samples. Astrocytes displayed similar trends, with 570 genes up-regulated and 146 down-regulated. Conversely, in the Double-Negative versus Intermediate comparison, excitatory neurons showed 4122 up-regulated and 552 down-regulated genes, while astrocytes had 1008 up-regulated and 331 down-regulated genes. Comparisons between Intermediate and Double-Positive groups revealed substantially fewer DEGs across all cell types analyzed (refer to Fig. 7, Supplementary Fig. 9, and Supplementary Table 5 for a full list of genes DE in each comparison). These findings align with another analysis where we correlated our data with 25 risk loci identified from a large genome-wide association meta-analysis of clinically diagnosed late-onset Alzheimer's disease (LOAD), involving 94,437 individuals [43]. As illustrated in Fig. 8*,* the analysis revealed that most of these genes exhibit differential expression patterns in excitatory neurons and astrocytes when comparing the Double-Negative versus Intermediate groups, as well as the Double-Negative versus Double-Positive groups.

### Consistent pathway perturbations in tau-PLA positive groups (Double-Positive and Intermediate) compared to Double-Negative group through enrichment analysis

**Fig. 9:**
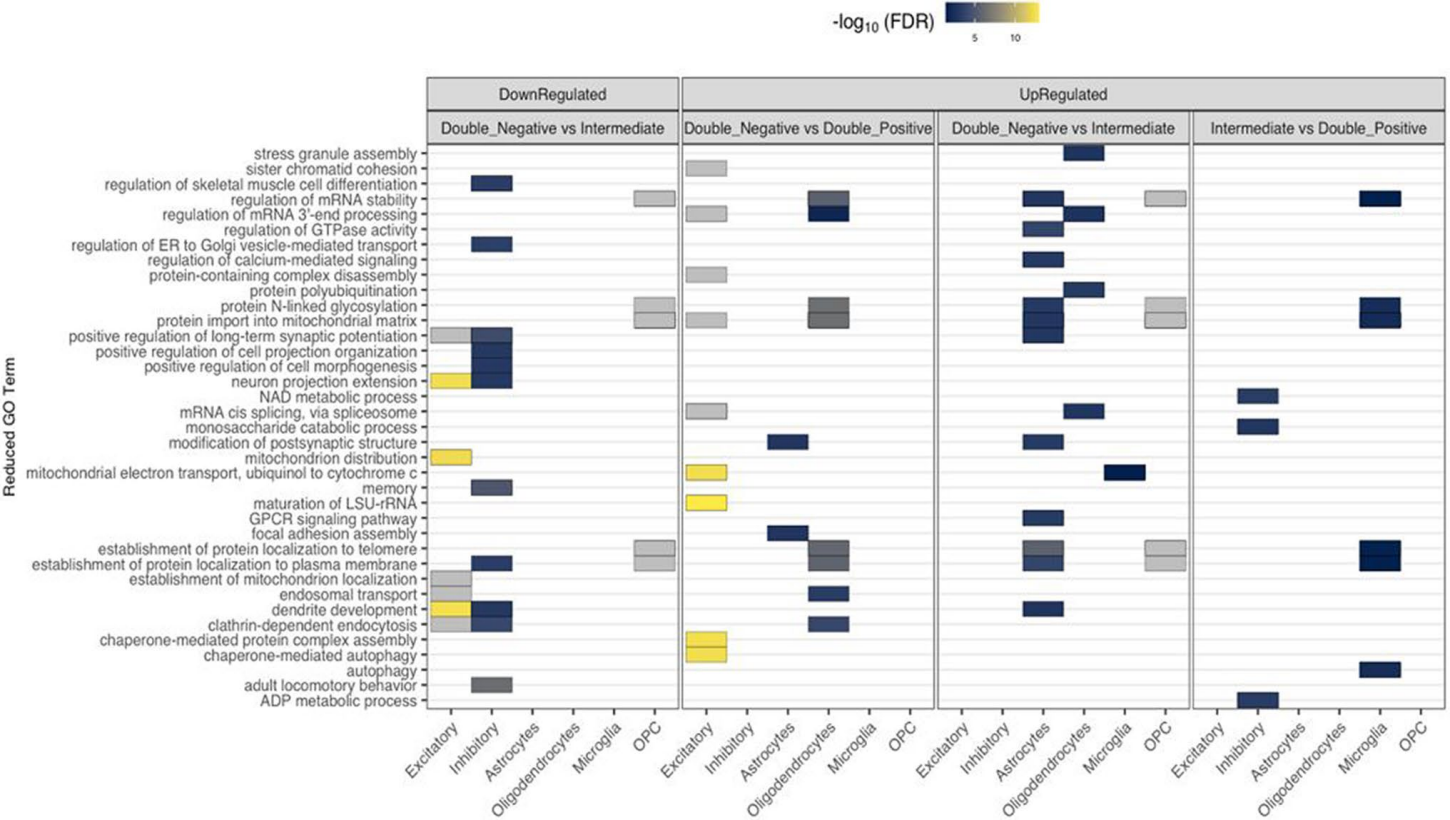
Significantly (FDR < 0.05) enriched pathways identified using DEG sets from each pairwise comparison. The fill of each tile indicates the − log10FDR value of the most significant child term associated with the parent term. The full list of child GO terms assigned to each parent term across all pairwise comparisons in the panel is available in Supplementary Table 6

To associate DEG sets with downstream functional consequences, over-representation analysis was carried out using the R package WebGestaltR [69]. In total, 472 BP GO (child) terms were enriched for DEG sets derived from Double-Negative versus Double-Positive comparison, while 594 and 162 GO (child) terms were enriched for Double-Negative versus Intermediate and Intermediate versus Double-Positive, respectively. As shown in Fig. 9, several overlapping pathways were identified between the Double-Negative versus Double-Positive and Double-Negative versus Intermediate comparisons, suggesting consistent pathway perturbations in tau-PLA positive groups (Double-Positive and Intermediate) compared to the Double-Negative group. These pathways include regulation of mRNA stability, regulation of mRNA 3'- end processing, protein-N linked glycosylation, protein import into mitochondrial matrix, establishment of protein localization to telomere, establishment of protein localization to plasma membrane, and clathrin-dependent endocytosis. Notably, in the Double-Negative versus Double-Positive comparison, the genes associated with these pathways were predominantly up-regulated in excitatory neurons and oligodendrocytes. In contrast, in the Double-Negative versus Intermediate comparison, these genes were primarily up-regulated in astrocytes and OPCs, while being predominantly down-regulated in OPCs. Conversely, none of the down-regulated genes appeared among the significantly enriched pathways in the Double-Negative versus Double-Positive and Intermediate versus Double-Positive comparison. For more details on BP pathways across different cell types, refer to Supplementary Table 6.

### Enrichment of heritability analysis

**Fig. 10:**
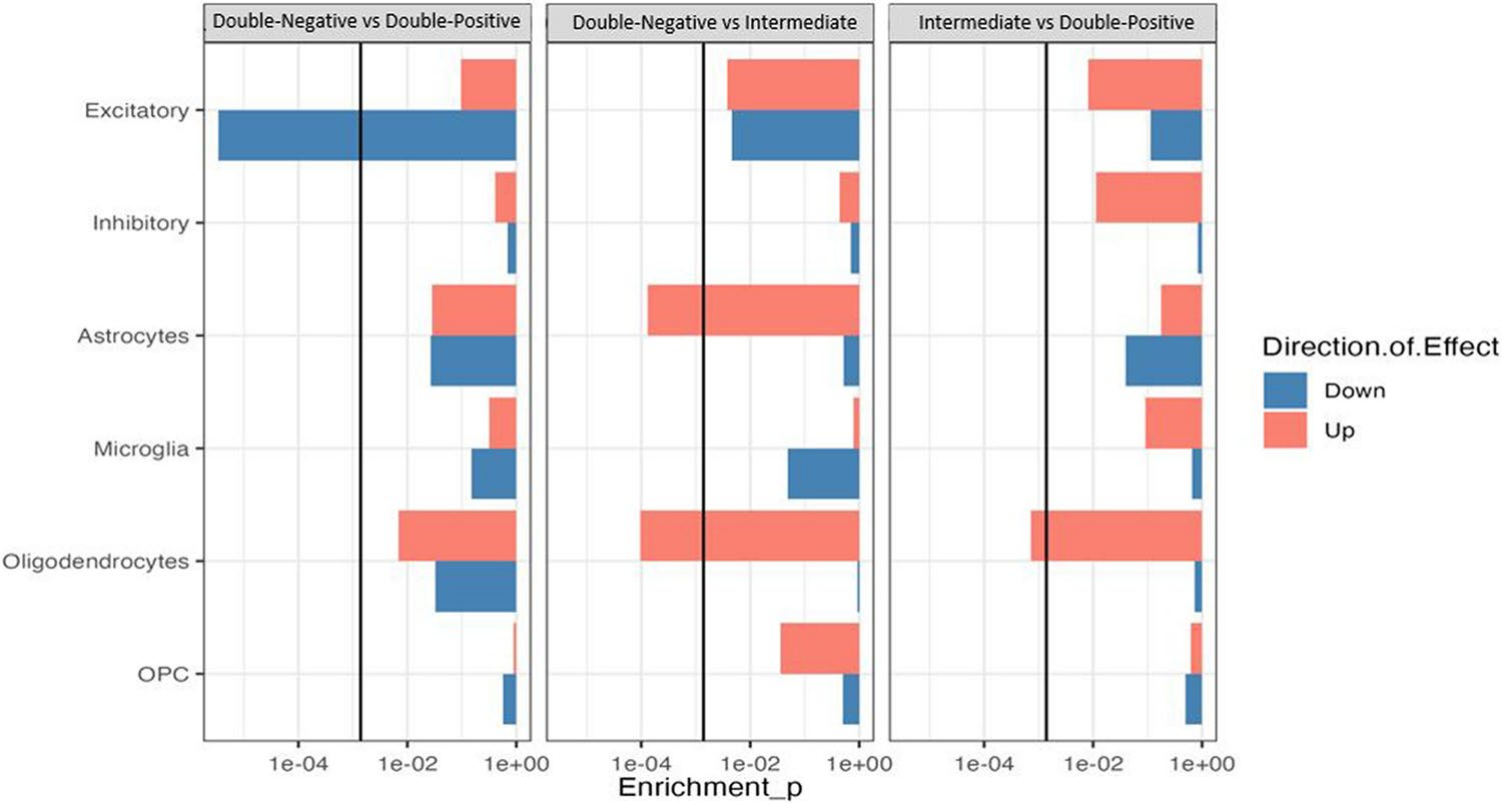
Genetic association with cell-type specific differentially expressed genes across all pairwise comparisons. LDSC was used to identify associations. The *x*-axis indicates enrichment *p*-values. The black line indicates Bonferroni significance threshold (*p*-values adjusted for the number of cell types tested; FDR < 0.05). The color of the bars indicates if the DEGs were up- or down-regulated. Full results can be found in Supplementary Table 7

To test the extent to which genes DE in the presence of visible multimeric tau plus or minus AT8-phosphorylated tau, as detected by tau-PLA and AT8-IHC, respectively, are enriched for a genetic association to AD, we utilized LDSC (Fig. 10). AD genetic risk genes exhibited a strong association with down-regulated genes in excitatory neurons (FDR_LDSC_ = 6.08E−05) in Double-Negative versus Double-Positive samples. In the comparison between Double-Negative and Intermediate, a significant (FDR < 0.05) association was found between AD risk genes and up-regulated genes in both oligodendrocytes (FDR_LDSC_ = 1.76E−03) and astrocytes (FDR_LDSC_ = 2.25E−03). Furthermore, a significant association was identified in the Intermediate versus Double-Positive comparison group between the AD risk genes and up-regulated genes in oligodendrocytes (FDR_LDSC_ = 1.15E−02). For a comprehensive overview of analysis statistics, refer to Supplementary Table 7.

### Early activation of reactive astrocytes was observed in the Intermediate group

Using Monocle 3, we successfully pseudotime-ordered astrocytic nuclei, uncovering dynamic changes in gene expression along the trajectory of the pathological categories (Double-Negative > Intermediate > Double-Positive; Supplementary Fig. 10). Notably, marker genes associated with reactive astrocytes, such as *C3, CHI3L1, FABP7,* and *GFAP* displayed significant alterations along this trajectory. Reactive astrocytes (A1), known to be activated in response to pathological stimuli in the central nervous system [46, 54], exhibited a distinct molecular profile (refer to Supplementary Fig. 10), suggesting an early involvement of reactive astrocytes in the formation of tau aggregates, particularly evident in the tau-PLA positive groups (Double-Positive and Intermediate). To evaluate if early astrocytic transcriptional changes were associated with different astrocytic morphology, we quantified GFAP expression in the grey and white matter of the temporal cortex across groups using GFAP-IHC. However, no statistically significant differences in GFAP% area were detected (Supplementary Fig. 11).

## Discussion

Despite decades of research, the understanding of early cellular changes in AD remains elusive, creating challenges in developing effective disease-modifying or preventive therapies. Recent investigations have shed light on the toxic nature of early tau multimers, implicating them in the propagation of tau pathology [36, 44, 53, 64], and tau multimers have been shown to appear from early stages in the medial temporal lobe [9]. In this study, we evaluated the development of tau multimerization, phosphorylation, and conformational changes alongside the Braak tau pathway beyond the medial temporal lobe. We then measured the tau seeding capacity and single nuclei transcriptomic changes of tissue samples differentially affected by tau molecular alterations. FFPE sections from temporal and occipital cortices of 67 cases across Braak stages 0 to VI were investigated by tau-PLA and tau-IHC using a panel of antibodies: AT180, T217, AT8, PHF-1, S422, S198, aS199, MC1, and Alz50 [2, 10, 26, 45, 68]. Diffuse tau-PLA signal was present from Braak stage I in the hippocampal regions and the inferior temporal cortex, from Braak stage II in middle and superior temporal cortices, from Braak stage III in parastriate occipital cortex, and from Braak stage IV in the striate occipital cortex, often without corresponding tau-IHC signals (Supplementary Fig. 3). As we show in Fig. 2, AT180 and T217 antibodies were overall the most effective in capturing these small tau aggregates, although with lower sensitivity than tau-PLA, aligning with previous reports [26]. AT8 detected small tau structures from Braak stage I in the entorhinal cortex, from stage III in the temporal cortex and from stage V in the occipital cortex. This supports the hypothesis that tau self-interaction is among the earliest detectable events in AD-type tau pathology, as echoed by other studies focused on tau's seeding ability [36, 39, 52]. However, the staining pattern of tau-PLA and tau-IHC showed minor differences for large tau lesions in later Braak stages (Supplementary Fig. 4). Antibodies like PHF-1 and S422 presented reduced sensitivity for the large tau lesions compared to the other markers, followed by the conformational antibodies MC1 and Alz50 that detected fewer tau aggregates in each anatomical brain region. Although tau-PLA recognized large fibrillar aggregates similarly to AT8-IHC, its sensitivity was somewhat reduced, which is likely due to PLA visualizing only half of all antibodies pairs successfully bound to a target.

Tau-tau interaction is a requisite of prion-like tau seeding. Therefore, after uncovering previously unrecognized diffuse tau pathology in the temporal and occipital lobes, we further explored the pathophysiological impact and progression of early-type tau multimers in AD using tau seeding amplification assays. RT-QuIC, a well-established seed amplification assay, has been previously applied to assess the seeding capacity of tau aggregates in AD [40, 59]. In our analysis, temporal cortex samples exhibiting diffuse tau pathology but lacking large tau lesions (Intermediate group) demonstrated elevated tau seeding capacity and a shortened RT-QuIC reaction lag phase, with values comparable to those observed in the Double-Positive group (Fig. 4). CSF samples from the Intermediate group also exhibited significantly higher seeding activity compared to the Double-Negative group (Supplementary Fig. 7). However, a direct comparison with the Double-Positive group was not possible, as no CSF samples were available from this group. While the RT-QuIC assay is a powerful tool for investigating the pathophysiological role of aggregated tau, it lacks spatial resolution. In contrast, in situ tau-PLA enables the visualization of species containing tau-tau interacting molecules, providing insights into their subcellular, cell-type-specific, and supracellular localization. Given the strong correlation observed in this cohort between in situ tau-PLA and tau RT-QuIC signals, combining these two approaches presents a promising strategy for a more comprehensive understanding of tau-tau interactions and their contribution to the progression of tau pathology in AD.

Moreover, a recent study utilizing the FRET biosensor assay—a complementary tau seeding amplification technique—has demonstrated increased tau seeding activity in brain regions where tau pathology had previously been undetectable with conventional staining methods, such as IHC [39]. To corroborate the findings from the RT-QuIC assay, we integrated the FRET biosensor cells into our study. This involved exposing HEK293T tau RD-CFP/YFP cells to the same brain homogenates used in the RT-QuIC assay. Results from both assays were congruent, revealing seeding activity in both Intermediate and Double-Positive samples (Fig. 5). Overall, these findings are consistent with previous studies indicating that tau seeding preferentially relies on a soluble oligomeric form of tau rather than fibrillar tangles [24], although tangles may also contain seeding-competent species, or may be capable of releasing active oligomers.

Interestingly, as demonstrated in Fig. 6, immunodepletion with the AT8 antibody significantly reduced tau seeding activity not only in the Double-Positive samples, but also in the Intermediate group, which contained an increased amount of diffuse tau multimers but a negligible number of NFTs. Given our findings that tau seeding parallels more strongly with the amount of diffuse multimeric tau than with the overall quantity of tangles, this suggests that the predominant seeding-competent species in the Intermediate group correspond to diffuse tau multimers which are AT8-phosphorylated, or at least a subset of the tau in them is. Their diffuse nature and low abundance in the Intermediate group may explain why they were not significantly detectable by AT8-IHC in the temporal cortex. Notably, however, the T217 antibody identified small-sized/diffuse tau-stained structures in this region, supporting the idea that incipient phosphorylation of diffuse multimeric species occurs in the Intermediate group. It is also worth noting that the rolling circle amplification step used in tau-PLA enhances the detectable size of each multimeric detected signal, which could explain why diffuse, relatively low-abundant oligomers are observed with tau-PLA but not as clearly with IHC, particularly if only a fraction of tau in the multimer pool is phosphorylated at this stage. Moreover, a previous study has shown that AD patients exhibit variable responses to different anti-tau antibodies in immunodepletion experiments, with no single antibody completely abolishing seeding activity [24]. Nevertheless, AT8 was among the most effective antibodies at reducing seeding activity, likely due to its ability to recognize phosphorylated, seeding-competent oligomers [24], in agreement with our results. The incomplete suppression of seeding activity observed with tau5 immunodepletion in our study may be explained by potential saturation of the immunodepletion process by the total tau pool and partial inhibition of tau5 binding due to tau phosphorylation, as highlighted in a recent study [25]. This suggests that tau5 may only bind a proportion of certain phosphorylated tau species, allowing certain seeding-competent multimers to escape immunodepletion. Regardless, the significant inter-individual variability in tau seeding activity [24] underscores the need for cautious interpretation of our findings and highlights the importance of using larger cohorts in future studies to validate these observations.

In this study, we scrutinized gene expression alterations associated with the earliest detectable abnormal tau species at the single-cell level. DEA illuminated shared and distinct transcriptional profiles between NFTs and early detected tau multimers. Notably, examination of the Intermediate group compared to the Double-Negative group revealed differential expression of numerous well-known genes associated with adult-onset neurodegenerative disorders. Examples include *APP, LRRK2, PINK1, SNCA, MAPT, PSEN1, PSEN2,* and *PARK7*, with most of these genes exhibiting no differential expression in comparisons between the two tau-PLA positive groups (Double-Positive and Intermediate). Furthermore, fewer genes displayed differential expression between the two tau-PLA positive groups (Double-Positive and Intermediate), indicating transcriptional similarities (Fig. 7). To validate these results, we performed an additional analysis correlating our data with 25 risk loci identified from a comprehensive genetic meta-analysis, as shown in Fig. 8 [43]. This analysis indicated that most of these genes display differential expression patterns in excitatory neurons and astrocytes [43]. For example, the expression of *CD2AP*, a gene that encodes a scaffold protein crucial for actin remodeling and membrane trafficking, is up-regulated in excitatory neurons when comparing both Double-Negative versus Double-Positive and Double-Negative versus Intermediate groups (Fig. 8). This observation is substantiated by recent studies which have revealed a strong association of *CD2AP* with AD progression and its colocalization with phosphorylated tau. Additionally, the distribution of *CD2AP* in neurons demonstrates a hierarchical pattern that correlates with the Braak neurofibrillary stage, irrespective of age and other neuropathological features [16].

Another gene, *PICALM,* has also been identified as a critical susceptibility gene for AD [3]. However, its expression patterns in AD are a subject of ongoing investigation. While some research indicates a reduction in *PICALM* expression in AD, potentially affecting endocytosis and the clearance of protein aggregates in the brain [70], other studies have observed an increase in *PICALM* mRNA levels in both the brain and blood of AD patients [8, 42, 63]. Our analysis mirrors these varied findings, revealing an up-regulation of *PICALM* in excitatory neurons in the Double-Negative versus Double-Positive group (Fig. 8). In contrast, a down-regulation is seen in astrocytes and inhibitory neurons in the Double-Negative versus Intermediate comparison (Fig. 8). These conflicting observations underscore the complexity of AD and the variety of factors influencing gene expression.

Another interesting finding is the downregulation of the *CLU* gene in astrocytes in the Double-Negative versus Intermediate group (Fig. 8). A study conducted to determine the role of astrocytic *CLU *in vivo has shown that *CLU* overexpression in astrocytes enhances excitatory neurotransmission, providing evidence for an essential role of *CLU* secreted from astrocyte in protection against AD by enhancing synaptic transmission [19].

By performing pathway enrichment analysis, we identified consistent pathway perturbations in tau-PLA positive groups (Double-Positive and Intermediate) compared to the Double-Negative group. As demonstrated in Fig. 9, only a few pathways appeared among significantly enriched pathways identified using DEG sets from each pairwise comparison in the Intermediate versus Double-Positive comparison, suggesting similar transcriptional profiles between these groups. In contrast, several pathways, such as regulation of mRNA stability, protein import into the mitochondrial matrix, and establishment of protein localization to plasma membrane, overlapped between the Double-Negative versus Double-Positive and Double-Negative versus Intermediate comparisons (Fig. 9).

The regulation of mRNA stability is critical for maintaining proper protein levels in cells, especially in neurons, where precise gene expression is essential for normal function. In AD, dysregulation of mRNA stability can lead to the production of abnormal proteins, such as increased levels of Aβ or tau, contributing to disease progression [32]. Interestingly, our study showed that genes involved in the regulation of mRNA stability were enriched primarily in glial cells, rather than neurons (Fig. 9). In astrocytes, RNA stability may be important for regulating the synthesis of proteins involved in neurotransmitter uptake, synaptic modulation, and neurotrophic support [58]. Impairment in this pathway can lead to synaptic dysfunction, contributing to neuronal damage observed in the early stages of AD. Furthermore, dysregulated RNA-binding proteins in astrocytes can influence inflammatory responses, exacerbating neurodegeneration [58]. In terms of oligodendrocytes, RNA stability is required for the proper synthesis of myelin-related proteins [35]. Disruption of this pathway can impair myelination and lead to axonal degeneration, further contributing to the cognitive decline associated with AD. Studies suggest that RNA-binding proteins in oligodendrocytes are critical for controlling the translation of myelin proteins, and their dysfunction may result in myelin deficits [35]. Similarly, OPCs rely on RNA stability for cell proliferation, differentiation, and myelination. Disruption in RNA stability within OPCs can impair their regenerative capacity, hindering remyelination efforts in neurodegenerative diseases like AD [35].

The mitochondrial protein import pathway was also enriched in both the Double-Positive and Intermediate groups compared to the Double-Negative group across neurons and glial cells. This finding aligns with research highlighting mitochondrial dysfunction as a key feature of AD [4, 7, 15]. Proper protein import into the mitochondrial matrix is essential for maintaining mitochondrial function and energy production. However, mitochondrial dysfunction can result in oxidative stress and reduced ATP production, accelerating neurodegeneration by depriving neurons of the energy required for maintenance and survival [4, 7, 15]. This dysfunction is commonly observed in the early stages of AD, where it is associated with synaptic failure and energy deficits, contributing to early cognitive decline.

Another shared pathway between the Double-Negative versus Double-Positive and Double-Negative versus Intermediate groups was protein localization to the plasma membrane. The interactions of tau with cellular membranes are crucial for intracellular signaling and neuronal functions [60]. Tau also interacts with membrane proteins and lipid tails which facilitate membrane translocation and form stable protein-lipid complexes involved in cell-to-cell transport [60]. However, in the disease, tau monomers can form reversible condensates at the membrane surface, alter the secondary structure, and induce oligomers, which can lead to irreversible crosslinking and fibril formation [60]. Furthermore, internal plasma membranes act as critical platforms for enzyme interactions and amyloid precursor protein (APP) processing, ultimately leading to the generation of Aβ peptides. In our analysis, most genes associated with this pathway were also expressed in glial cells, suggesting their crucial role in both the formation of tau multimers and the induction of their toxic effects.

The fact that most overlapping pathways between Double-Negative versus Intermediate comparison groups were up-regulated in astrocytes suggests the involvement of astrocytes in early AD-type tau pathology. Several genes widely expressed in astrocytes (e.g., *APOE-ε4, WWOX, CLU*, and *CDK2AP1*) are related to AD risk and progression and have been implicated in tau metabolism [57]. Activation of specific astrocyte pathways could be crucial in promoting either neuroprotective or deleterious effects in response to tau [38, 46, 62]. Further supporting this, we observed a high expression of marker genes for A1 astrocytes in tau-PLA positive (Double-Positive and Intermediate) groups and performed GFAP-IHC analysis (Supplementary Fig. 10 and Supplementary Fig. 11). However, the absence of statistically significant differences in the percentage of GFAP-positive stained areas across the groups may indicate that transcriptional and functional changes in reactive astrocytes precede morphological changes. Moreover, accurately quantifying GFAP stains is challenging due to their complex shapes, underscoring the need for further investigation into the interplay between tau oligomers and astrocytic activation. Refined super-resolution microscopy techniques may be required to reveal possible fine morphological changes in astrocyte cell bodies and projections associated with early transcriptomic changes. Additionally, it is important to note that GFAP staining may not capture all reactive astrocytes, potentially underestimating astrocytic responses.

This study is not without limitations. The use of post-mortem tissue prevents us from establishing the clinical trajectory of individuals and the precise temporal sequence of pathological events, which limits our ability to establish causality. For example, we cannot establish whether diffuse tau-PLA pathology progresses into NFTs, as alternative explanations are theoretically possible. For instance, diffuse tau-PLA could be an early marker of cellular alterations that later contribute to tau neurofibrillary aggregation. It is also conceivable that small multimers diffuse from localized large lesions. However, if this were the case, we would expect a gradient of tau-PLA, with the highest levels around large neurofibrillary lesions or in regions near or connected to NFT-rich areas, but this was not obviously observed. Another possibility is that diffuse tau pathology is a normal part of aging or even a mechanism of resilience, activated in parallel to pathological events. Additionally, we cannot entirely rule out the possibility that cases from the Double-Positive group may represent instances of resilient AD. Although case reports were carefully examined, the retrospective nature of our study limits our ability to fully exclude this possibility. We also acknowledge that cases from the Double-Negative and Intermediate groups might correspond to early stages of Primary Age-Related Tauopathy (PART) rather than AD. However, distinguishing between these conditions in the early stages is challenging, as both exhibit very low or undetectable amyloid beta pathology [11, 12].

In designing effective AD transcriptional analyses, this study underscores the limitations of relying solely on the presence of large protein aggregates for group stratification. Instead, the presence of small tau multimers and tau seeding capacity should be taken into account. We show that early-stage tau multimerization—occurring ahead of the formation of NFTs—is sufficient to drive tau seeding and associate distinct transcriptomic signatures overlapping with those found in tissue harboring numerous NFTs. While future research is needed to establish if these early-stage gene expression and tau seeding changes represent a potentially reversible “first hit” or if pathological cascades are already committed, the overall modest effect of therapies targeting late-type pathological species highlights that more research should be focused on early molecular events as those we describe here.

## Supplementary Information

Below is the link to the electronic supplementary material.

Supplementary file1 (PDF 837 KB)

Supplementary file2 (XLSX 32 KB)

Supplementary file3 (XLS 2783 KB)

Supplementary file4 (CSV 302 KB)

Supplementary file5 (XLS 28 KB)

## Data Availability

The raw and processed sequencing data generated in this study have not yet been deposited. All scripts used in this study can be found in GitHub Supplementary materials including full tables https://github.com/rahfel/tauPLA.
