# Supplementary material for: Seeding-competent early tau multimers are associated with cell type-specific transcriptional signatures
Source: Acta Neuropathol. 2025 Apr 4;149(1):31. doi: 10.1007/s00401-025-02869-4 (PMC11971191; doi:10.1007/s00401-025-02869-4)
Supplement: Supplementary file 1 — Supplementary file1 (PDF 837 KB) [file 401_2025_2869_MOESM1_ESM.pdf]

Seeding-competent early tau multimers are associated with cell type-specific transcriptional signatures

Simona Jogaudaite#[1], Rahel Feleke#[1], Elisavet Velentza-Almpani#[1], Leung Yeung-Yeung[1], Daniel Clode[1], Jeong Hun Ko[1], Maria Otero-Jimenez[1], Ben Shin[1], Steve Matthews[1], Sarah J. Marzi[1,2,3], Maxwell P. Spires-Jones [4], Tara L. Spires-Jones[4], Michael R. Johnson[1], Javier Alegre-Abarrategui[1]

# These authors contributed equally to this work

1 Department of Brain Sciences, Imperial College London, Hammersmith Hospital, London, W12 0NN, UK

2 UK Dementia Research Institute at King's College London, London, SE5 9RT, UK

3 Department of Basic and Clinical Neuroscience, Institute of Psychiatry, Psychology and Neuroscience, King's College London, London, SE5 9RT, UK

4 The University of Edinburgh Centre for Discovery Brain Sciences and UK Dementia Research Institute, Edinburgh, EH8 9JZ, UK

Correspondence to:

Javier Alegre-Abarrategui

j.alegre@imperial.ac.uk

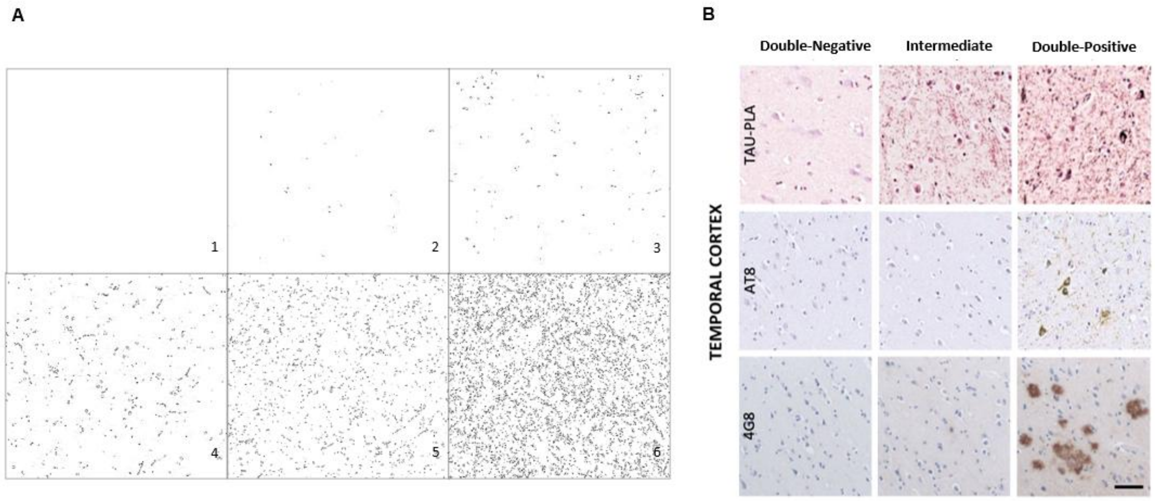

Supplementary Fig. 1 A Semi-quantitative scale used for analysis. **B** Representative images of temporal cortex from Double-Negative, Intermediate, and Double-Positive groups stained with tau-PLA, AT8-IHC, and 4G8-IHC. Scale bar 50 µm

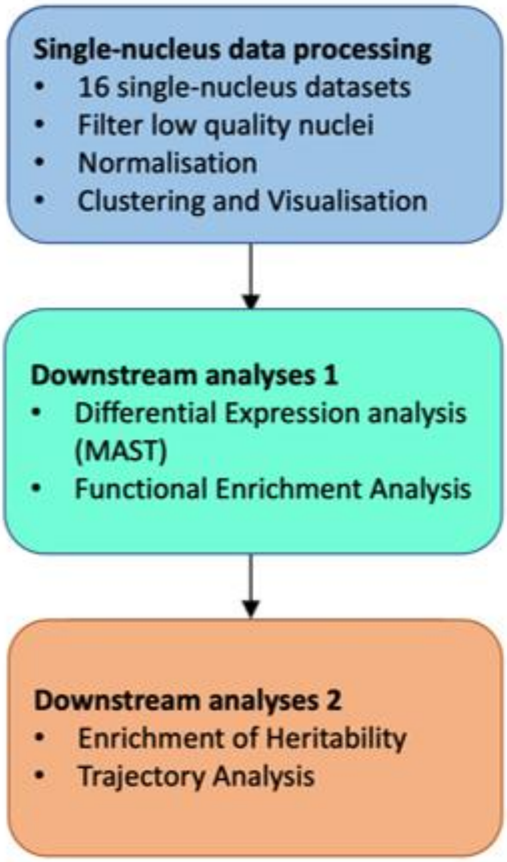

Supplementary Fig. 2 Overview of pre-processing steps and downstream analysis carried out using 16 human brain tissue samples

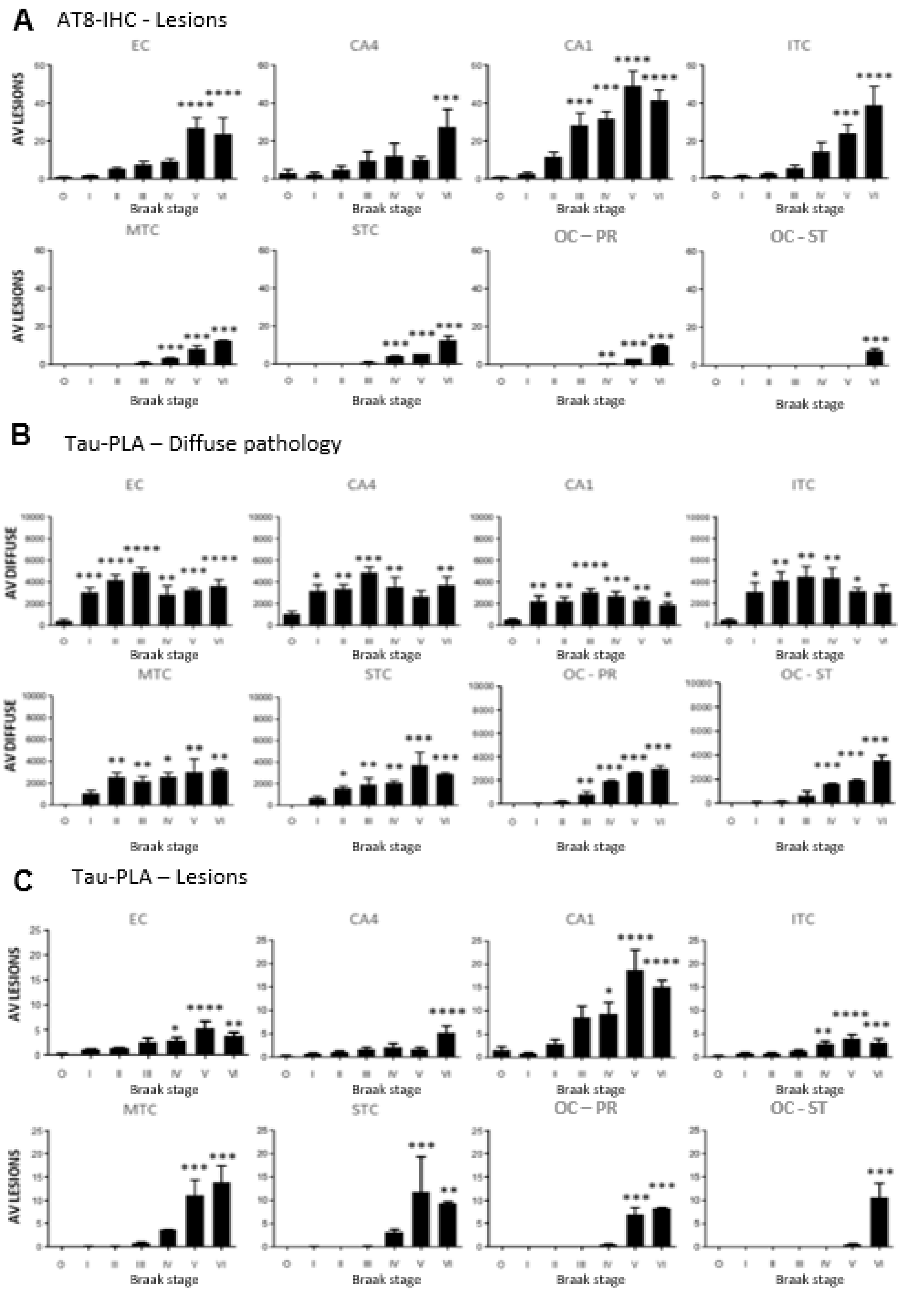

Supplementary Fig. 3 Quantification of AT8-IHC labeled large perikaryal neurofibrillary-type lesions and tau-PLA labeled diffuse pathology and lesions in entorhinal, temporal and occipital cortices. Automated quantification of **A** AT8-IHC labeled large lesions, **B** tau-PLA diffuse pathology, and **C** tau-PLA labeled large lesions across the different brain areas. EC, CA4, CA1, OC, PR, and ST data in this figure were reproduced from the previous publication (doi 10.1186/s40478-020-01117-y). All groups were compared to the control (Braak 0) through a One-way ANOVA (Dunnett); N = 11/12/12/9/7/8/8. Each bar represents the mean ±

standard error of the mean (SEM). *p < 0.05, **p < 0.01, ***p < 0.001, ****p < 0.0001. Abbreviations: *EC:* Entorhinal cortex, *ITC:* Inferior temporal cortex, *MTC:* Middle temporal cortex, *STC:* Superior temporal cortex, *OC:* Occipital cortex, *PR:* Parastriate area, *ST:* Striate area, *AV:* average

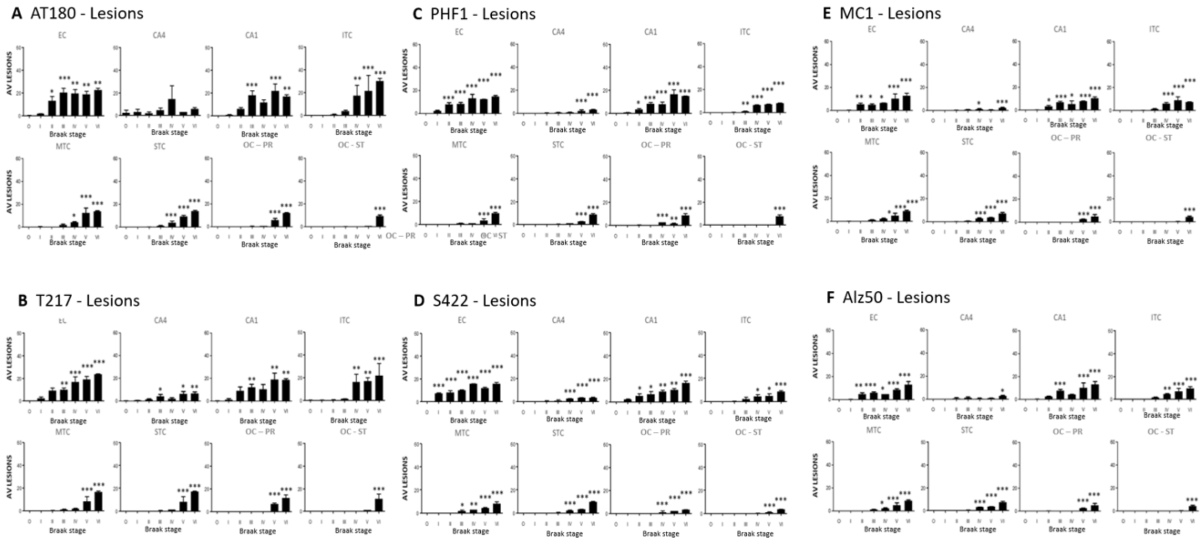

Supplementary Fig. 4 Quantification of AT180-, T217-, PHF-1, S422-, MC1- and Alz50-IHC labeled large perikaryal neurofibrillary-type lesions in the hippocampus, temporal and occipital cortices. Automated quantification of **A** AT180-, **B** T217-, **C** PHF-1-, **D** S422-, **E** MC1-, and **F** Alz50-IHC labeled large lesions across the different brain areas. All groups were compared to the control (Braak 0) through a One-way (Dunnett); N = 11/12/12/9/7/8/8. Each bar represents the mean ± standard error of the mean (SEM). *p < 0.05, **p < 0.01, ***p < 0.001, ****p < 0.0001. Abbreviations: *EC:* Entorhinal cortex, *ITC:* Inferior temporal cortex, *MTC:* Middle temporal cortex, *STC:* Superior temporal cortex, *OC:* Occipital cortex, *PR:* Parastriate area, *ST:* Striate area, *AV:* average

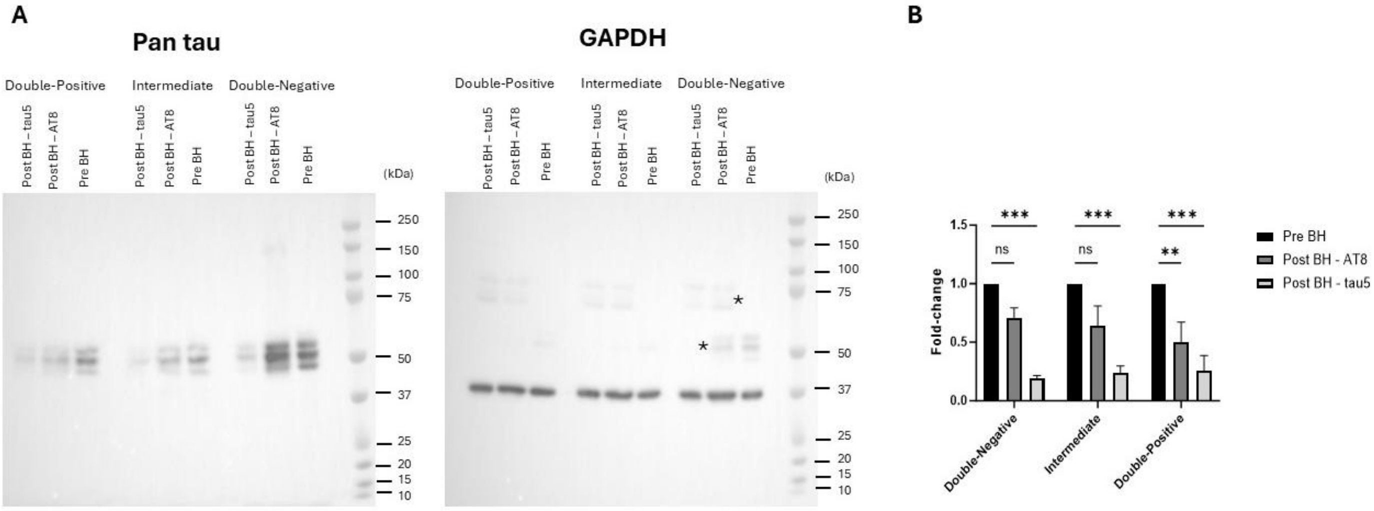

Supplementary Fig. 5 Western blot analysis of immunodepleted brain homogenates. A Detection of pan tau in non-immunodepleted (pre BH) and immunodepleted brain homogenates with AT8 antibody (post BH – AT8) and tau5 antibody (post BH – tau5) across Double-Negative, Intermediate, and Double-Positive groups. The blot was stripped and reprobed with GAPDH as a loading control to ensure equal protein loading across all samples. Asterisks (*) indicate bands corresponding to the immunoglobulins used for immunodepletion, detected due to being the same species as the anti-GAPDH antibody, and residual tau bands due to incomplete stripping. Molecular weight markers (kDa) are indicated on the right. **B** Representative fold changes in densitometry for pan tau protein levels using Western blot analysis. Each column represents a normalized ratio (fold-changes) to GAPDH and to control (non-immunodepleted brain homogenate, pre BH). One sample from each group was run in duplicates. Data was evaluated using Two-way ANOVA comparing to the control (pre BH) followed by Dunnett's Multiple Comparison Test. Each bar represents the mean ± standard error of the mean (SEM).

*$p < 0.05$, **$p < 0.01$, ***$p < 0.001$

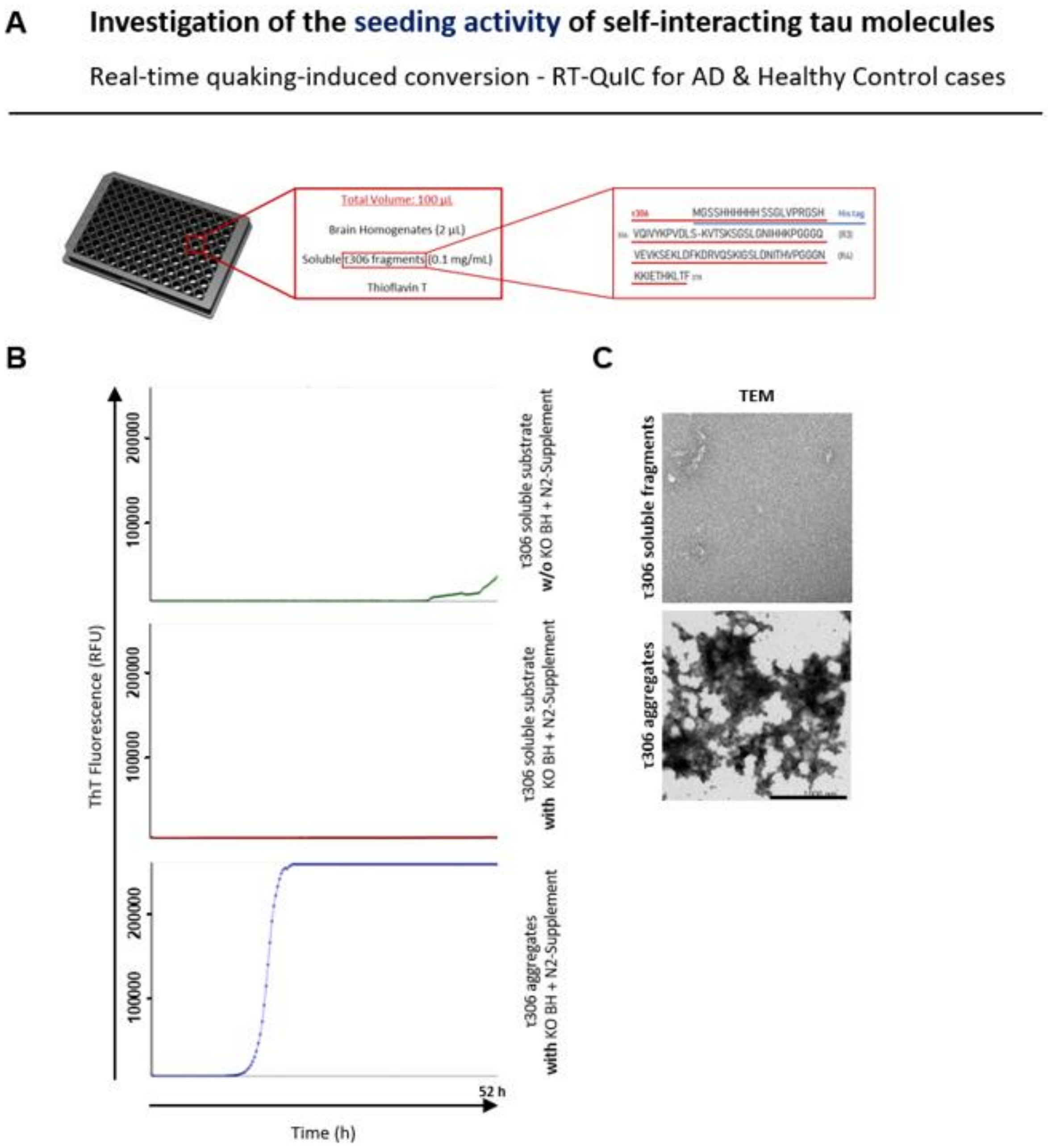

Supplementary Fig. 6 ThT fluorescence data of RT-QuIC analysis. **A** Schematic representation of RT-QuIC reaction and τ306 tau plasmid construct, consisting of residues 306–378 of full-length human tau isoform htau40 with a point mutation at residue 322 cysteine to serine. **B** RT-QuIC analysis of τ306 soluble fragments and τ306 aggregates under different conditions. Each curve represents a single case, run in triplicate for each dilution. **C** Transmission electron microscopy of recombinant τ306 soluble monomers and τ306 aggregated species. Aggregation of recombinant τ306 fragment was induced by shaking in the presence of heparin. Scale bar 1000 nm. Abbreviations: *TEM:* Transmission electron microscopy

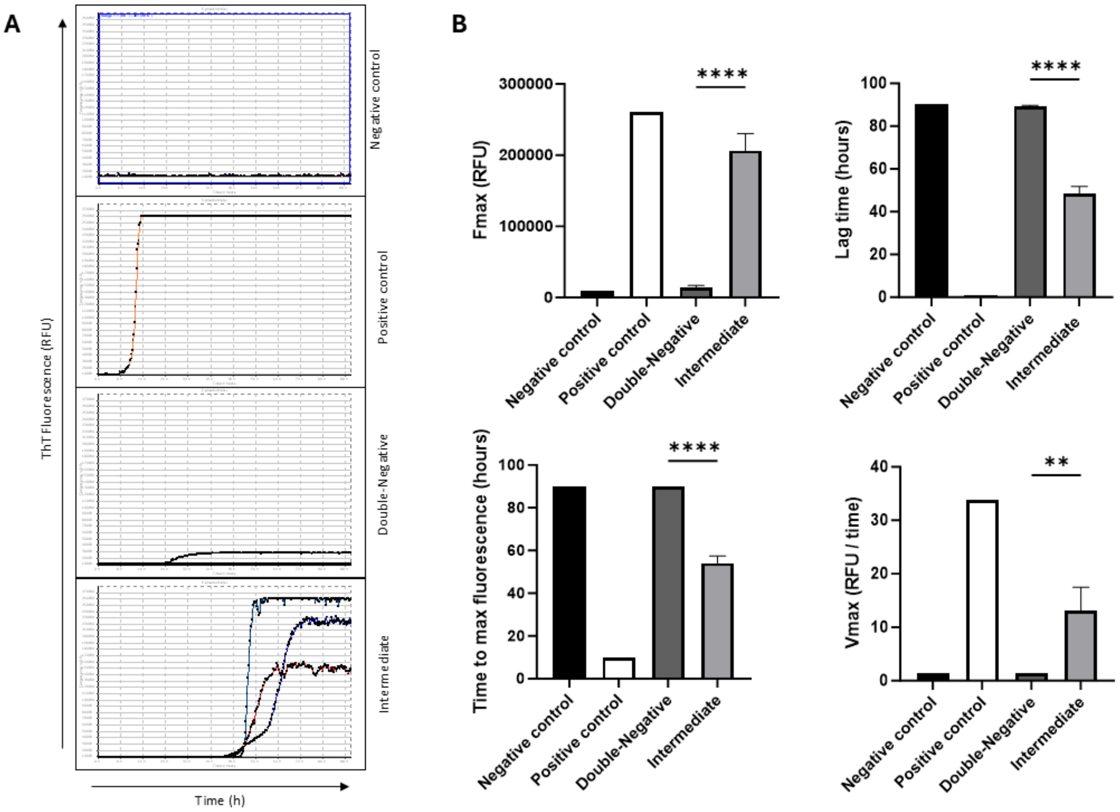

Supplementary Fig. 7 Tau RT-QuIC analysis using CSF samples. **A** RT-QuIC analysis of negative control (tau MAPT KO mouse brain homogenate), positive control (τ306 aggregates), Double-Negative and Intermediate CSF. Each curve represents a single case, run in triplicate. **B** Comparison of tau seeding activity of negative control (tau MAPT KO mouse brain homogenate), positive control (τ306 aggregates), Double-Negative, and Intermediate CSF with RT-QuIC. Fmax (maximum ThT fluorescence), lag time (reaction time to exceed a ThT fluorescence threshold of the average baseline fluorescence + 5 SD), time to reach maximum ThT fluorescence, and Vmax (maximum slope) were analyzed. The assay cut-off was determined to be 90 h as a reproducible endpoint before the spontaneous amyloid aggregation in the negative control wells. Groups were assessed through unpaired t test; N = 3/3. Each bar represents the mean ± standard error of the mean (SEM). $*p < 0.05$, $**p < 0.01$, $***p < 0.001$, $****p < 0.0001$

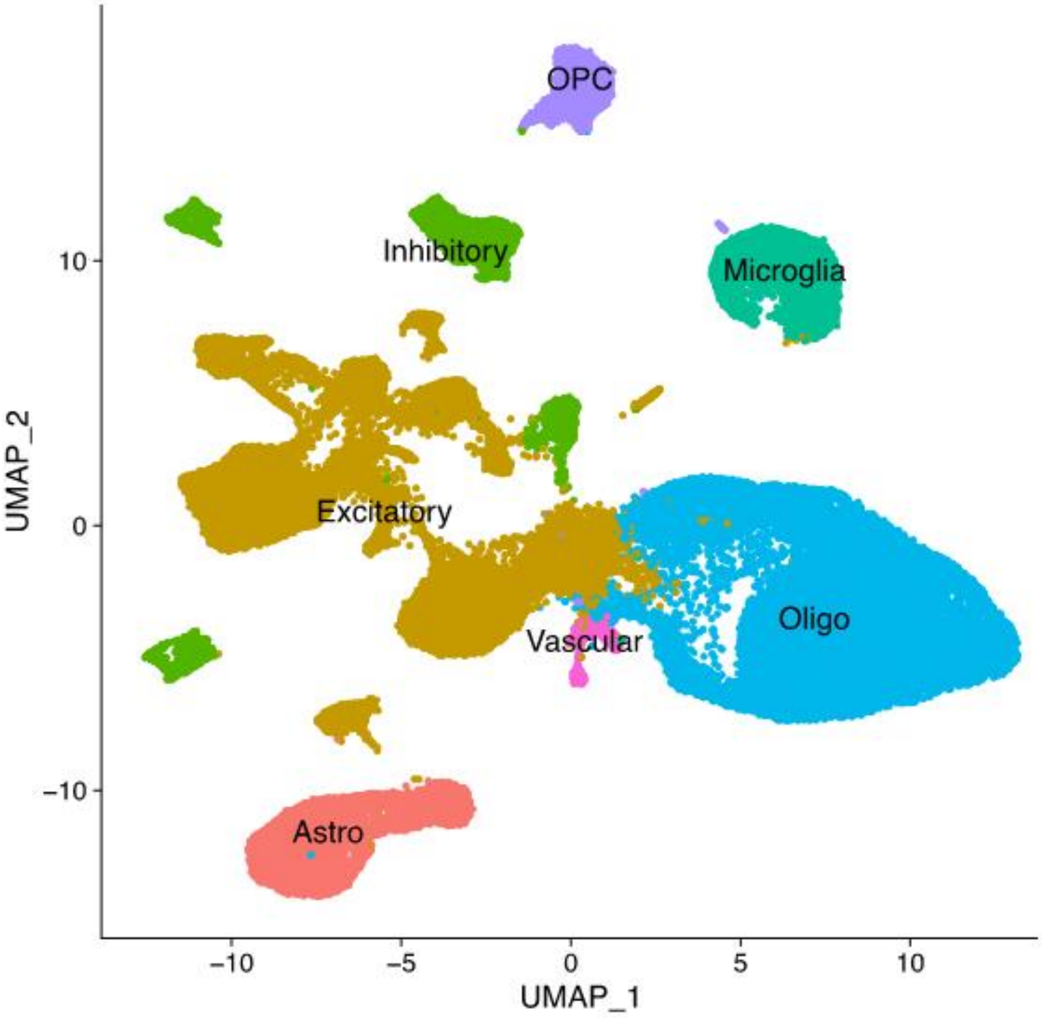

Supplementary Fig. 8 A Uniform Manifold Approximation and Projection (UMAP) plot illustrating nuclei derived from all subjects, with a joint plot created by amalgamating around 52,000 nuclei. Abbreviations: *Oligo*: Oligodendrocytes, *OPC*: Oligodendrocyte Progenitor Cells, *Astro:* Astrocytes

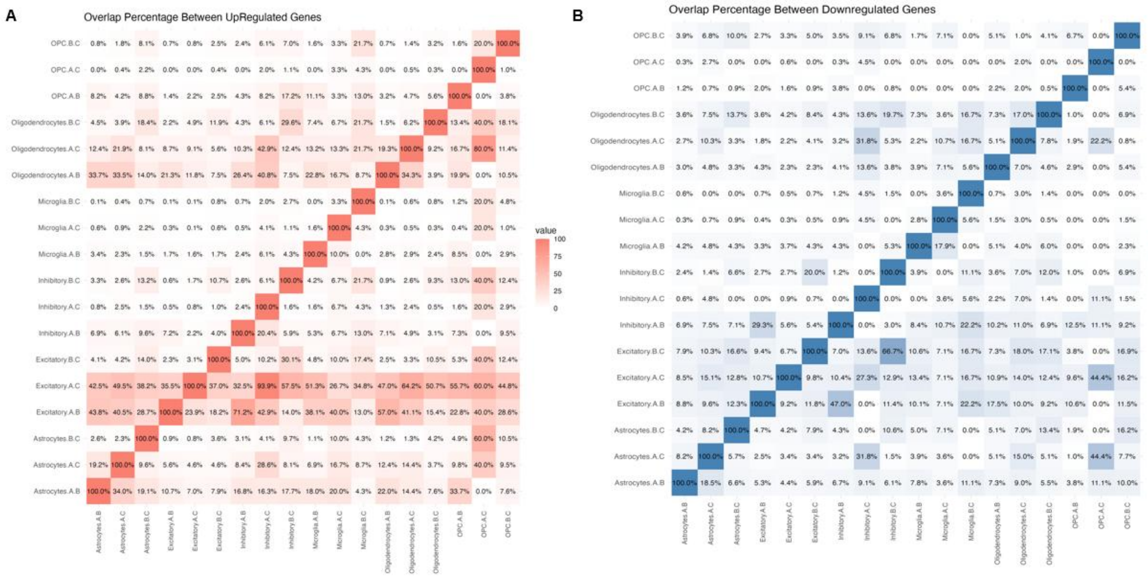

Supplementary Fig. 9 Percentages of overlapping DEGs across all comparisons. **A** Red colored squares for up-regulated genes. **B** Blue colored for down-regulated genes. *Abbreviations: A:* Double-Negative; *B:* Intermediate; *C:* Double-Positive

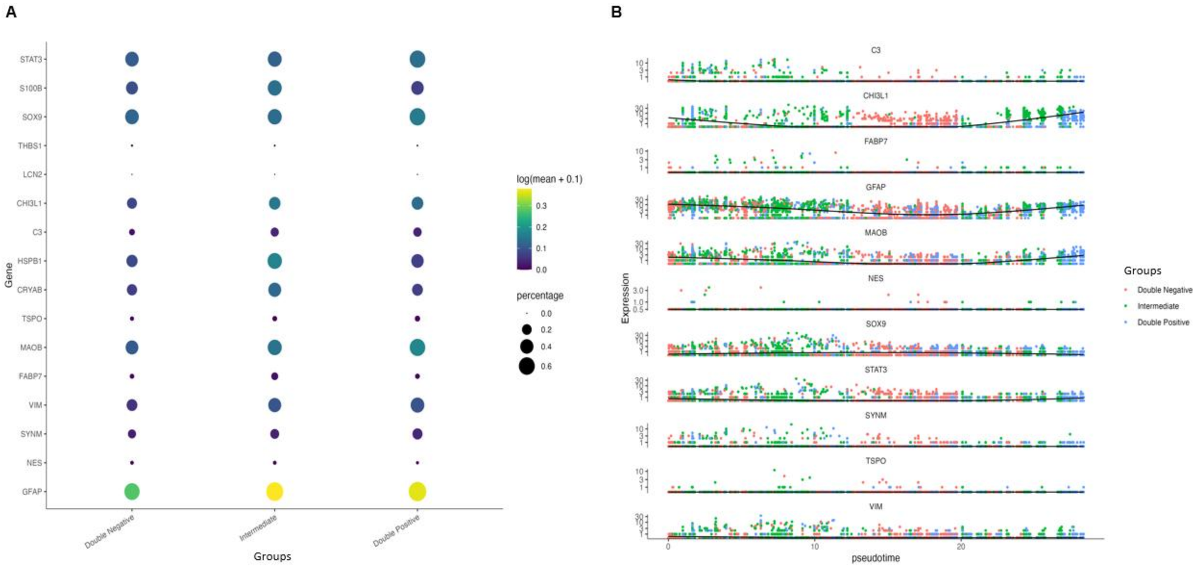

Supplementary Fig. 10 Expression levels of reactive astrocyte marker genes across the groups. **A** Log mean expression values of marker genes in each group. **B** Pseudotime plot of marker genes along a trajectory (from Double-Negative to Double-Positive)

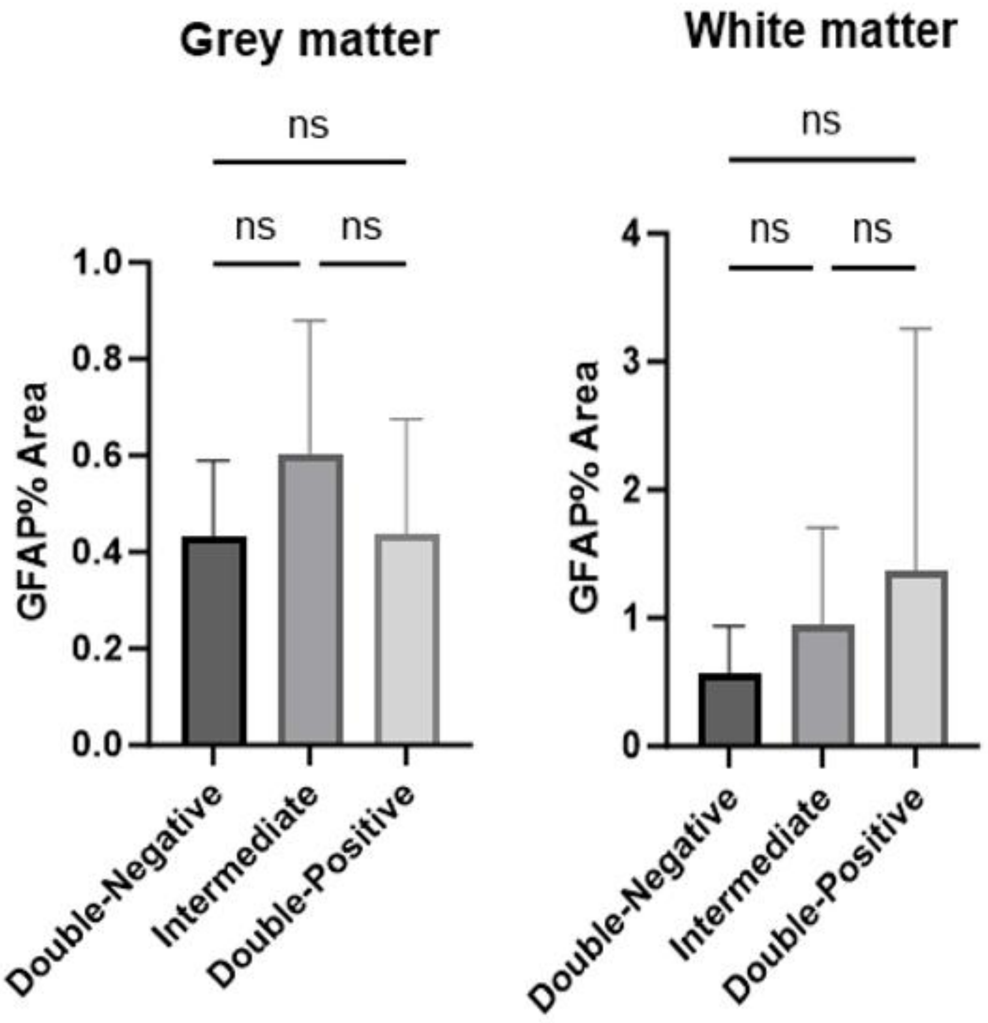

Supplementary Fig. 11 Immunohistochemical staining of GFAP in the temporal cortex. The percentage of GFAP-positive stained area was assessed in both the white and grey matter regions of the temporal cortex across three distinct groups: Double-Negative, Intermediate, and Double-Positive. Groups were assessed through a One-way ANOVA (Bonferroni); N = 6/5/5/. Each bar represents the mean ± standard error of the mean (SEM).
